# The cyclic peptide G4CP2 enables the modulation of galactose metabolism in yeast by interfering with GAL4 transcriptional activity

**DOI:** 10.3389/fmolb.2023.1017757

**Published:** 2023-03-01

**Authors:** Stefano Rosa, Andrea Tagliani, Chiara Bertaso, Luca Tadini, Cristina Visentin, Louise Jane Gourlay, Sabrina Pricl, Lucia Feni, Sara Pellegrino, Paolo Pesaresi, Simona Masiero

**Affiliations:** ^1^ Department of Biosciences, Università degli Studi di Milano, Milan, Italy; ^2^ Molecular Biology and Nanotechnology Laboratory (MolBNL@Units), DEA, University of Trieste, Trieste, Italy; ^3^ Department of General Biophysics, University of Łódź, Łódź, Poland; ^4^ DISFARM-Department of Pharmaceutical Sciences, University of Milan, Milan, Italy

**Keywords:** GAL4, cyclic peptide, combinatorial library, yeast two-hybrid, protein interference, galactose metabolism, drug discovery

## Abstract

Genetically-encoded combinatorial peptide libraries are convenient tools to identify peptides to be used as therapeutics, antimicrobials and functional synthetic biology modules. Here, we report the identification and characterization of a cyclic peptide, G4CP2, that interferes with the GAL4 protein, a transcription factor responsible for the activation of galactose catabolism in yeast and widely exploited in molecular biology. G4CP2 was identified by screening CYCLIC, a Yeast Two-Hybrid-based combinatorial library of cyclic peptides developed in our laboratory. G4CP2 interferes with GAL4-mediated activation of galactose metabolic enzymes both when expressed intracellularly, as a recombinant peptide, and when provided exogenously, as a chemically-synthesized cyclic peptide. Our results support the application of G4CP2 in microbial biotechnology and, additionally, demonstrate that CYCLIC can be used as a tool for the rapid identification of peptides, virtually without any limitations with respect to the target protein. The possible biotechnological applications of cyclic peptides are also discussed.

## Introduction

Over the last decades, peptides have been shown to be effective bioactive molecules, with a wide range of applications such as building blocks for synthetic biology or active compounds for drugs and antimicrobials (Fosgerau and Hoffmann, 2015; Rafferty et al., 2016; Zorzi et al., 2017; de la Torre and Albericio, 2020; Magana et al., 2020; Muttenthaler et al., 2021; Rosa et al., 2021; 2022). Peptides gained an increasing interest as they can provide specific binding of the target proteins (Tavassoli, 2017; Vinogradov et al., 2019; Sohrabi et al., 2020), their production is compatible with both synthetic and recombinant DNA methodologies (Fuse et al., 2018; Jaradat, 2018; Zhang et al., 2018; Cook and Pfleger, 2019; Vassaux et al., 2019; Cheng and Hua, 2020; Mejía-Pitta et al., 2021) and, they can be designed *de novo* *in silico* (Fletcher et al., 2012; Thompson et al., 2012; Langan et al., 2019; Chen Z. et al., 2020; Cao et al., 2020; Chen and Elowitz, 2021; Mulligan et al., 2021; Bhardwaj et al., 2022). Moreover, peptides can be considered as a class of compounds between small- and macro-molecules (Sohrabi et al., 2020; Apostolopoulos et al., 2021) as they display some favorable features belonging to both classes. As small molecules, peptides can be chemically modified to improve their solubility, specificity, and affinity toward their target, as well as their resistance to proteases (Vinogradov et al., 2019). Furthermore, they possess the ability, typical of macromolecules, to specifically disrupt protein-protein interactions (PPIs), preventing the formation of functional protein complexes (Contini et al., 2017; Dapiaggi et al., 2017; Marcelli et al., 2019; Sohrabi et al., 2020). This is of particular interest when the target protein lacks classical “druggable pockets”—i.e. catalytic or allosteric sites—as in the case of transcription factors (TFs) (Henchey et al., 2010; Ramaswamy et al., 2018; Henley and Koehler, 2021).

In fact, more than 80 peptide-based therapeutics are currently available on the market and their sales exceed 50 billion U.S.$, i.e. about 5% of the global pharmaceutical market (Muttenthaler et al., 2021). Peptides have also displayed their potential as antimicrobials for pest control and have been proposed as alternative/integrative compounds to conventional pesticides for disease-control strategies in agriculture, granting a lower environmental impact (Järvå et al., 2018; Schwinges et al., 2019; Colombo et al., 2020; Velivelli et al., 2020; Huang et al., 2021; Rosa et al., 2022).

DNA-encoded peptide libraries enable the simultaneous screening of millions or billions of peptides at the same time, in cell-based or *in vitro* assays, enabling the rapid identification, through DNA sequencing, of peptide sequences able to specifically interact and, possibly, influence the activity of target proteins (Tavassoli, 2017; Sohrabi et al., 2020; King et al., 2021). In this context, cyclic peptides (CPs)—i.e. “peptidic structures bearing a ring that spans multiple amino acid residues” (Vinogradov et al., 2019)—are preferable to their linear counterparts for many reasons, as they display higher *in vivo* stability, improved resistance to exo- and endo-peptidases and structural rigidity (Bucci et al., 2020), which favour tighter and more target binding (Tapeinou et al., 2015; Sohrabi et al., 2020; Muttenthaler et al., 2021; Zhang and Chen, 2022). Moreover, peptide cyclization might enhance cellular permeability, allowing efficient interactions with intracellular targets (Bockus et al., 2013; Kelly et al., 2021; Mendive-Tapia et al., 2021).

Several genetically-encoded cyclic peptide libraries have been developed using phage display (Desimmie et al., 2012; Wang et al., 2019; Chen S. et al., 2020; Simonetti and Ivarsson, 2020), mRNA-display (Yamagishi et al., 2011; Goto and Suga, 2021) or the split-intein circular ligation of peptides and proteins (SICLOPPS) (Scott et al., 1999; 2001; Tavassoli and Benkovic, 2007). The latter method exploits trans-splicing split-intein domains belonging to DNA polymerase III (*DnaE*) from *Synechocystis* sp. PCC6803, and the possibility of inserting the peptide libraries between the two permutated intein domains (I_C_ and I_N_). Upon intein auto-processing, the peptide is head-to-tail cyclized and released intracellularly (Scott et al., 2001; Tavassoli and Benkovic, 2007). SICLOPPS was successfully employed in many cell-based assays, exploiting prokaryote (Tavassoli et al., 2008; Nordgren and Tavassoli, 2014) and eukaryote (Kritzer et al., 2009; Mistry and Tavassoli, 2017) organisms. Modified versions of SICLOPPS have also been developed to generate lariat peptides. Lariat refers to cyclized peptides having an ester bond between a side-chain hydroxyl group and the C-terminus. By substituting an asparagine residue with an alanine (N36A) in the Ic subunit of the SICLOPPS construct, intein-mediated cyclization is arrested at an intermediate step, resulting in cyclic peptides retaining an exposed N-terminus bridging the newborn lariat to the I_C_ domain (Barreto et al., 2009; Barreto and Geyer, 2014). Therefore, this SICLOPPS variant can be fused to a module of interest, like the bacterial LexA transcriptional repressor (Barreto et al., 2009), making it suitable for Yeast Two-Hybrid (Y2H) assays, which enable the identification of peptides that interact with a protein target of interest (Paiano et al., 2019).

In this study, we used the SICLOPPS lariat-generating cyclic peptide strategy to obtain a combinatorial cyclic peptide library suitable for the GAL4-based Y2H assay (Fields and Song, 1989). The advantage of using the Y2H system relies on the fact that the screening can be performed *in vivo* with no need to express and purify the target protein. This technology was used to screen for CPs that can physically interact with the GAL4-DNA Binding Domain (GAL4-DBD). Among the several GAL4-DBD-interacting cyclic peptides (G4CPs) identified, G4CP2 is shown, by means of activity assays *in vivo*, to interfere with GAL4 transcriptional activity when supplied both intra- and extracellularly to a GAL4-harbouring strain, proving that our technology can be adopted for multiple purposes, including metabolic engineering (Chen and Elowitz, 2021; Rosa et al., 2021).

## Materials and methods

### Plasmids, yeast strains and growth conditions

The CYCLIC library was constructed in the pGADT7-KanMX vector, a derivative of pGADT7 (Clontech), which was modified by inserting the KanR resistance gene under the control of TEF1 promoter and terminator (KanMX cassette). Unless stated otherwise, all cloning procedures were performed using the DNA synthesis and cloning services at Officinae Bio (Venice, Italy). CYCLIC-harboring plasmids were transformed into the AH109 (*MATa, trp1-901, leu2-3, 112, ura3-52, his3-200, gal4Δ, gal80Δ, LYS2::GAL1*_*UAS*_ *-GAL1*_*TATA*_ *-HIS3, GAL2*_*UAS*_ *-GAL2*_*TATA*_ *-ADE2, URA3::MEL1*_*UAS*_ *-MEL1*_*TATA*_ *-lacZ*) yeast strain (Clontech). GAL4-DBD was expressed using the pGBKT7-GW plasmid (kindly provided by Prof. Brendan Davies) transformed into the Y187 (*MATα, ura3-52, his3-200, ade2-101, trp1-901, leu2-3, 112, gal4Δ, met* ^*–*^ *, gal80Δ, URA3::GAL1*_*UAS*_ *-GAL1*_*TATA*_ *-lacZ*) yeast strain (Clontech). A construct for producing a linear version of G4CP2 was generated by homologous recombination, co-transforming NruI-linearized pGADT7-KanMX-SspIntein plasmid with a PCR-reconstituted fragment in yeast, using 8xNNK flanking Fw, 8xNNK flanking_Rev and G4CP2-stop oligonucleotides (Supplementary Table S1). In this construct, we introduced a stop codon between the G4CP2 sequence and the Intein_N_ element.

GAL4 reporter activity assays and phenotypic assays were carried out in the S288C-derivative strain BY4741 (*MATa, his3Δ1, leu2Δ0, met15Δ0, ura3Δ0*), transformed with the SLVD02 plasmid (Escalante-Chong et al., 2015)—*HO-GAL1p-YFP-hphNT1-HO* integration cassette—upon NotI linearization. GST-Intein ± G4CP chimeras were expressed by substituting the GAL4 Activation Domain (GAL4-AD) with a GST-tag coding sequence in the pGADT7-KanMX-SspIntein vector. BY4741 Δ*gal4*, Δ*gal1*, Δ*gal80* mutant strains were purchased from SRD—Scientific Research and Development GmbH (Oberursel, Germany). Yeast cells were cultured at 30°C both on solid and liquid media, unless otherwise stated in the text, untransformed strains were cultured on 2xYPDA and, when transformed with plasmids and/or integration cassettes, on SD media depleted of nutrient(s) required for auxotrophic selection and/or supplemented with antibiotic(s). 2% *(w/v)* glucose was used in media unless differently specified.

### Library construction and quality control

The SspIntein gene (Barreto and Geyer, 2014), was generated by annealing 8 overlapping oligonucleotides (Supplementary Table S1) and Klenow-mediated fill-in at 25°C for 8 h. The reconstituted gene was then amplified using flanking primers (Supplementary Table S1), digested with EcoRI and XhoI restriction enzymes and cloned into the pGADT7-KanMX vector, previously linearized with EcoRI and SalI. 50 µg of pGADT7-KanMX-SspIntein were linearized with NruI and de-phosphorylated. The oligonucleotide for library recombination (library fragment) in pGADT7-KanMX-SspIntein (Supplementary Table S1) was obtained by PCR, generating a 141 bp amplicon (Supplementary Figure S1). Library transformation was performed using the protocol described by Gietz and Schielts (2007), with minor modifications. The AH109 yeast strain was cultured overnight in 2xYPDA, diluted to 0.5 OD_600_ in a total 2xYPDA volume of 600 mL and grown for ∼5 h (∼2.0 OD_600_). The culture was harvested, extensively washed with water, pooled in a single 50 mL centrifuge conical tube, and resuspended with the transformation mix: 28.8 mL 50% *(w/v)* PEG3350, 4.32 mL 1 M lithium acetate, 6 mL single stranded DNA carrier (2 mg/mL), 48 µg of linearized plasmid, 5.4 mL of library fragment and 2.2 mL of DMSO. After vigorous vortexing, the yeast cell suspension was divided into several 2 mL microcentrifuge tubes and incubated at 42°C for 60 min. Cells were harvested and incubated in 2xYPDA at 30°C for 3 h. After collection, cells were plated on 150 Petri dishes (150 mm diameter) poured with SD–L (G418 200 μg/mL). Library titer was estimated plating serial dilutions of the pooled transformations. Additionally, small scale transformations, to estimate the number of transformed yeast cells without the CP-encoding sequence contained in the library, were also performed by comparing two small scale transformations with or without the library fragment (estimated number of library members without the CP insertion is 1 out of 609 library members). After 5 days of growth at 30°C, cells were harvested from plates, and the pellet was re-suspended at a 1:1 ratio with SD-L supplemented with 50% *(v/v)* glycerol and stored at −80°C, in 500 µL aliquots. The library titer was estimated after thawing a stored aliquot (3.6 x 10^6^ CFU/μL).

### Mating-based Y2H screening for the identification of GAL4-DBD-interacting (G4CPs) peptides

Y187 yeast cells transformed with pGBKT7-GW vector were mated with CYCLIC-expressing cells (AH109) following guidelines provided by Takara Bio USA (PT4084-1). Cells were plated on SD -W-L-H-A + 5 mM 3-AT (3-amino-1,2,4-triazole) + 200 μg/mL G418 (Geneticin) and incubated at 30°C for 7 days. CP-encoding sequences were identified using standard PCR procedures and amplicons were sequenced using the PlateSeq Kit PCR service (Eurofins Genomics). Only peptide-encoding sequences, correctly recombined in the SspIntein gene, were annotated. A consensus sequence search was performed with the algorithm developed by Dhanda et al. (2018), using “cluster-break” method and setting 50% identity threshold (Supplementary Table S2) and graphically represented using WebLogo3 (Crooks et al., 2004; Supplementary Figure S2).

### G4CPs interaction strength

**FIGURE 1:**
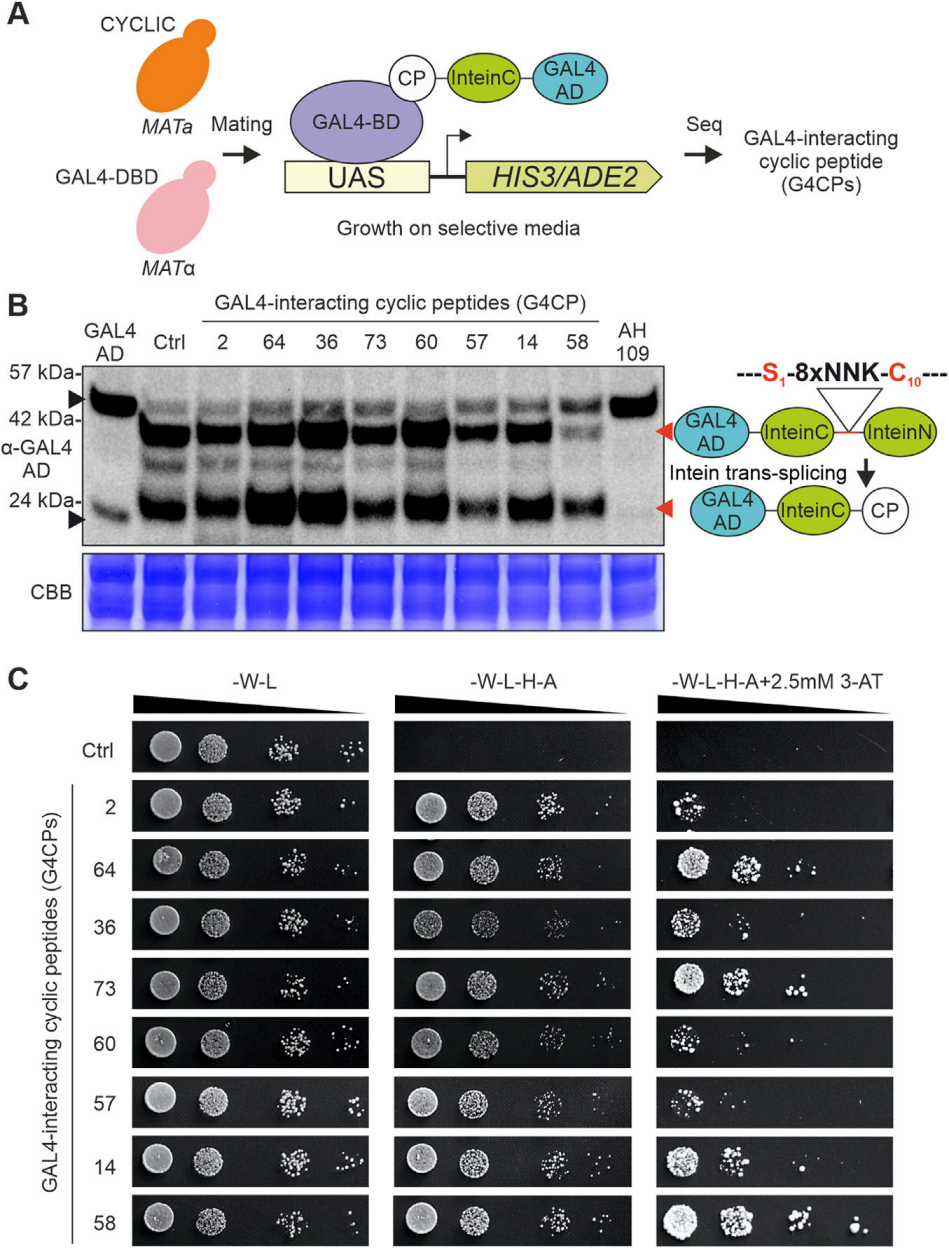
CYCLIC library validation. **(A)** Mating-based Yeast Two-Hybrid (Y2H) screening. CYCLIC was transformed into the AH109 (*MATa*) yeast strain, which was then co-cultivated with the compatible mating type Y187 (*MATα*) yeast strain; the latter was transformed with a GAL4-DNA Binding Domain (GAL4-DBD)-expressing plasmid, to identify bait-interacting peptides through Y2H assay, in the resulting diploid yeast cells. **(B)** Eight yeast clones, expressing G4CP-encoding constructs, were selected and their intein-mediated cyclization was assessed through western blot (WB) analysis, using a GAL4-AD primary antibody. All peptide sequences underwent cyclization, as shown by the presence of two bands at ∼38 kDa (upper red arrow) and ∼23 kDa (lower red arrow), respectively, corresponding to the full-length un-spliced protein (GAL4-AD-InteinC-8xNNK-InteinN) and to the spliced protein (GAL4-AD-InteinC-CP). As controls, the following samples were included: i) yeast cells transformed with the pGADT7-KanMX (GAL4-AD), which displays a signal with a slight shift in mass (∼21 kDa) (lower black arrow) respect to the spliced inteins (∼23 kDa), corresponding to the GAL4-AD alone; ii) yeast cells transformed with the pGADT7-KanMX-SspIntein vector (Ctrl), which performs intein splicing, even in the absence of 8xNNK sequence; iii) untransformed yeast cells (AH109), which demonstrates that the signals at higher molecular weight (>42 kDa) (upper black arrow)—appearing in all the analysed samples—are not related to GAL4-AD-Intein chimeras. It must be noted that the 8xNNK sequence is flanked by a Serine (Ser_1_) and a Cysteine (Cys_10_), belonging to InteinC and InteinN portions, respectively, which are required for intein-mediated splicing and peptide sequence cyclization. The protein bands visible between the linear and cyclic peptides represent cyclization intermediates. Experiment was carried out using AH109 yeast strain. CBB, Coomassie Brilliant Blue staining of the corresponding SDS-PAGE used as loading control. **(C)** The same eight selected yeast clones of panel B were assayed for their interaction strength with the GAL4-DBD, again in a Y2H assay. Optical densities of yeast cell cultures were normalized, serially diluted and spotted on different media. As can be observed on selective plates of increasing stringency (from -W-L-H-A to -W-L-H-A +2.5 mM 3-AT), the strains expressing different G4CPs display different growth levels. It is important to note that yeast strain expressing the control plasmid does not grow on selective media, highlighting the absence of interaction between the CYCLIC scaffold and GAL4-DB.

Plasmids encoding for G4CPs were isolated using the Macherey-Nagel NucleoSpin Plasmid mini-prep kit—following the manufacturer’s protocol after bead beating in Buffer A1—and used to transform *E. coli* DH10B cells. Plasmids recovered from bacteria were used to transform AH109 yeast strains following the lithium acetate protocol (Clontech user manual PT1172-1). Subsequently, transformants were mated with Y187 strain, harboring the pGBKT7-GW vector and plated on SD–W –L (G418 200 μg/mL). To assay the interaction strength between the identified G4CPs and the GAL4-DBD, cells obtained from the different matings were cultured in liquid medium, OD_600_ normalized to 0.5, serially diluted and spotted on SD–W –L (control) and SD–W –L–H –A (G418 200 μg/mL) with/without 3-AT (Figures 1A-C).

### Protein extraction, SDS-PAGE and western blotting

Total soluble proteins were extracted from overnight liquid yeast cultures either by NaOH treatment (Kushnirov, 2000) or bead-beating (Dunn and Wobbe, 2001) methods. The quantity of proteins loaded into SDS-PAGEs was standardized by measuring the OD_600_ of liquid cultures. For SDS-PAGE, mPAGE™ Bis-Tris Precast Gels (Merck Life Sciences) were used. After electrophoresis, proteins were transferred onto a PVDF membrane using the semi-dry Bio-Rad Transblotter system. After blocking in TBS-T buffer supplemented with 5% *(w/v)* skimmed milk (Sigma-Aldrich), the membranes were incubated with primary antibodies according to suppliers’ instructions. The anti-GFP and anti-GST antibodies were purchased from Invitrogen (Cat# A-11122) and GE Healthcare (GE27-4577-01), respectively; the anti-GAL4-AD antibody was purchased from Merck Life Science (G9293-200UG). Chemi-luminescence was detected using HRP-conjugated secondary antibodies and ECL substrate, and a Bio-Rad ChemiDoc Imaging System was used to visualize and record chemiluminescent signals.

### Pull-down interaction assay

GST-G4CP2 and GST-SspIntein-carrying yeast strains (AH109) were grown at 30°C overnight. Cells were then lysed in 500 µl Pull-down (PD) buffer [60 mM HEPES-KOH pH 8.0, 150 mM NaCl, 60 mM KOAc, 10 mM MgOAc, 0.3% NP40 *(v/v)*] and proteinase inhibitor cocktail (cOmplete™, COEDTAF-RO, Roche), supplemented with 4 U DNase I (Roche) and lyticase for 30 min at 28°C. After bead beating, the lysate was cleared by centrifugation (16,000 g, 15 min) and incubated for 2 h at 4°C with 60 µl of Glutathione Sepharose affinity chromatography resin (Cytiva 17–0756-01). Glutathione sepharose resin was then recovered by a centrifugation step (1,000 g, 5 min) and washed with PD buffer. BL21 (DE3) pLysS *E. coli* cells expressing 6xHis-GAL4-DBD (in the pET14b vector) were lysed in 500 µl PD buffer supplemented with 4 U DNase I (Roche) and lysozyme. The cell lysate was cleared (16,000 g, 15 min) and incubated for 90 min (4°C on a rotating wheel) with the GST-G4CP2- and GST-SspIntein-bound glutathione sepharose resin. To stabilize the interaction between GST-G4CP2 and 6xHis-GAL4-DBD, 0.75% *(v/v)* of formaldehyde (FA) was added to the samples. After 30 min incubation (4°C on a steering wheel), the FA-mediated crosslinking was quenched by the addition of 125 mM glycine and the sepharose resin was washed 3 times with PD buffer for 10 min each. The sepharose resin was then eluted with 100 µl 4xDTT-LDS sample buffer (Merck-Millipore) and incubated at 70°C for 15 min, to revert the FA crosslinking.

### Lithium toxicity assay

The lithium toxicity assay to monitor GAL4 activity was performed, as described in Masuda et al. (2008) in *S. cerevisiae* BY4741 *pGAL1::YFP* strain. Overnight yeast liquid cultures were grown in SD -L (2% *(w/v)* raffinose) supplemented with hygromycin (200 μg/ml) and G-418 (200 μg/ml), optical density standardized to 0.5 OD_600_ and serial dilutions were spotted on plates. Composition of SD -L plates for the lithium toxicity assay was 2% *(v/v)* glycerol, 0% or 2% *(w/v)* galactose and 0 or 40 mM LiCl. Yeast growth was monitored over 3-4 days.

### Peptide synthesis

The cyclic peptide G4CP2 (βA-RYFFDMWY) was chemically synthesized in-house, to tune the experimental conditions, and subsequently purchased from CASLO ApS (Denmark). G4CP2 was synthesized by microwave-assisted automated Fmoc/*t*Bu-based solid phase peptide synthesis (MW-SPPS) (Pellegrino et al., 2012) using Liberty Blue synthesizer (CEM Corporation). Chlorotrityl resin was used as solid support with a loading of 0.5 mmol/g and the synthesis was carried out on a 0.1 mmol scale. All amino acids were *N*-terminally Fmoc (fluorenylmethoxycarbonyl)-protected, while the side chains of trifunctional amino acids were protected with orthogonal, acid labile groups. The following side chain protecting groups were used: 2,2,4,6,7-pentamethyldihydrobenzofuran-5-sulfonyl (Pbf) for Arg, *tert*-Butyl (*t*Bu) for Asp and Tyr and tertbutyloxycarbonyl (Boc) for Trp. Coupling was performed using 5 equivalents (eq) of the protected amino acid, previously dissolved in dimethylformamide (DMF) to obtain a 0.2 M solution. As coupling reagents 5 eq of *N*,*N*′-Diisopropylcarbodiimide (DIC, 0.5 M in DMF) and 5 eq of Oxyma Pure (1 M in DMF with the addition of 0.1 M *N*,*N*-Diisopropylethylamine, DIPEA, to decrease the acidity of the solution) were used. To deprotect the Fmoc group, a solution of piperidine in DMF (20% *(v/v)* was applied. The coupling reaction was accomplished at 25°C for 120 s, followed by 480 s at 50°C and 35W. To couple arginine residues, a double coupling procedure was carried out. The Fmoc group was cleaved using a standard de-protection protocol at 75°C, 155 W for 15 s, followed by 60 s at 90°C, 50 W. The resin was treated with a solution of dichloromethane:trifluoroethanol:acetic acid 8:1:1 for 2 h at room temperature (RT). The solution was then filtered and transferred to a flask. The solvent was evaporated under reduced pressure, and the peptide was precipitated in water and freeze-dried. After freeze-drying, the product was subjected to the cyclization step (Feni et al., 2020; Feni and Neundorf, 2022). 1 eq of the full protected linear peptide was dissolved in DMF (0.2 mM); consequently, benzotriazol-1-yloxytripyrrolidinophosphonium hexafluorophosphate (PyBOP, 6 eq) and 1-Hydroxybenzotriazole (HOBt, 6 eq) were added. DIPEA was added till the solution reached pH 8. The reaction was left with stirring at RT. After 6 h, PyBOP was added again, and the reaction was left stirring overnight. The day after, the solvent was evaporated by reduced pressure, the crude peptide was diluted with ethyl acetate and extracted with brine and saturated NaHCO_3_. The organic phase was then dried at reduced pressure and subjected to full cleavage. The crude peptide was treated with TFA/phenol/water/thioanisole/3,6-dioxa-1,8-octanedithiol (82.5:5:5:5:2.5, 3 ml in total). The reaction was continuously stirred at RT for 3 h. Afterwards, the mixture was put in a vial containing 30 ml of cold diethyl ether (Et_2_O) in order to precipitate the peptide. The mixture was centrifuged and washed five times with cold Et_2_O. The crude peptide was freeze-dried and dissolved in H_2_O:acetonitrile (ACN) 65:35 + 0.1% trifluoroacetic acid (TFA) and purified on semipreparative reversed-phase high performance liquid chromatography, RP-HPLC (Jasco PU-2086, Adamas C18-Classic 10 µ, 250 × 21.2 mm ID). Acetonitrile/water with 0.1% TFA were used as eluents with a gradient of 35%–80% ACN in 40 min. The peptide was freeze-dried from water obtaining a white solid (20% yield). ESI-MS (m/z) C_65_H_79_N_13_O_14_S_1_: calculated, 1280.5; found 1280.4.

CP11 (βA-ELRYSSIP) was purchased from CASLO ApS (Denmark).

### Fluorescence measurements

The fluorescence signal of BY4741 *pGAL1::YFP* reporter strain was measured using a Varioskan LUX multimode microplate reader (ThermoFisher Scientific). Overnight cultures were pelleted, washed twice in water and OD_600_ normalized for the different cultures. An equal volume of normalized cultures was pipetted in each well of a 96-well plate containing different galactose/glucose (Gal/Glu) ratios. When treating the cells with chemically synthesized G4CP2, a 10 mM stock solution (100% DMSO) was diluted to a working concentration of 100 µM in each well designated for treatment. Fluorescence and OD_600_ measurements were performed by incubating plates at 28°C, shaking 2 min at 180 rpm, before each measurement.

Each experiment was at least repeated twice, and statistics analyses were performed using GraphPad Prism 8.

### RNA extraction, cDNA synthesis and quantitative real-time PCR

BY4741 *pGAL1::YFP* yeast strain expressing GST-Intein (Control) or GST-G4CP2 were grown o/n in SD -L 2% (*w/v*) raffinose, supplemented with G418 (200 μg/μL) and Hygromycin (200 μg/μL). Cells were harvested and washed in liquid YSD medium, inoculated in SD -L 1% (*w/v*) glucose or galactose (supplemented with G418 200 μg/μL and Hygromycin 200 μg/μL) and cultured for 1 h at 30°C. Cell pellets were resuspended in 800 µL of acidic phenol and 800 µL of extraction buffer [100 mM Tris-HCl pH 7.5, 100 mM LiCl, 10 mM EDTA, 1% *(w/v)* SDS], incubated for 45 min at 65°C and vortexed every 10 min. After cooling, microtubes were centrifuged for 2 min at 15,000 rcf at 4°C, and the upper aqueous phase was washed twice with an equal volume of chloroform. After LiCl precipitation (4 M final concentration), the RNA pellet was washed twice with EtOH 75%, dried and resuspended in nuclease-free water. RNA was quantified using NanoDrop One (ThermoFisher Scientific). 1 µg of total RNA has been retro-transcribed using iScript™ gDNA Clear cDNA Synthesis Kit (Bio-Rad Laboratories S.r.l.), following manufacturer guidelines. Real time (RT)-qPCR was performed using iTaq universal SYBR Green Supermix (Bio-Rad Laboratories S.r.l.) and primers for *GAL1*, *GAL2*, and *ACT1*, the latter as normaliser (Han and Emr, 2011). Relative expression of target genes was quantified using CFX Connect Real-Time PCR Detection System (Bio-Rad Laboratories S.r.l.). For each analyzed target gene, the RT-qPCR was repeated twice, where each repetition included four independent biological replicates for each yeast strain and condition analyzed. Statistical analysis was performed using GraphPad Prism 8.

### *In vitro* passive membrane permeability of G4CP2

The passive permeability of G4CP2, along with a further set of 27 compounds, was assessed using the parallel artificial membrane permeation test (PAMPA-BBB) (Di et al., 2003) following an established protocol (Estrada-Valencia et al., 2019; Campora et al., 2021). Accordingly, a semiautomated pipetting device (BenchSmart 96, Mettler Toledo) and a microplate spectrophotometer (SpectraMax Plus 384 microplate reader, Molecular Devices) were used for pipetting and UV reading, respectively. The porcine brain lipid (PBL, catalogue n. 141101C) was purchased from Avanti Polar Lipids, the Millex filter units (PVDF membrane, 0.45 μM pore size) were acquired from Millipore, while commercial compounds and reagents were obtained from Sigma-Aldrich. A 96-well acceptor microplate (PTFE, Millipore) was prepared by filled each well with 300 μL of PBS/DMSO (95:5, pH = 7.4), while the donor microplate artificial membrane (PVDF membrane, pore size 0.45 m, Millipore) was carefully coated with 5 μL of PBL dissolved in dodecane (20 mg/L). Each compound was dissolved in DMSO, diluted with PBS/DMSO (95:5, pH = 7.4) to a final concentration of 40–100 μM in the donor well, filtered with a Millex filter, and applied to the donor microplate wells (200 μL). Next, the donor plate was carefully placed on top of the acceptor plate, thereby bringing the artificial membrane in contact with the buffer solution underneath. The donor well was covered with a lid, and the whole system was left undisturbed overnight (18 h) at 25°C in a container sealed with damp paper towels to prevent evaporation. After incubation, the donor microplate was carefully removed, and UV-vis spectroscopy was used to determine the amounts of the tested compounds in the acceptor and donor microplate wells, respectively. Each sample was evaluated at five wavelengths in four wells during three separate runs; accordingly, all data are presented as mean values with relevant standard deviation. The permeability values (P_e,exp_, cm/s) were calculated according to the following expression: P_e,exp_ = {−V_d_V_a_/[(V_d_ + V_a_)At]ln–1—d_a_/d_eq_}, in which V_d_ and V_a_ represent the volumes of the donor and the acceptor wells, respectively, A is the surface of the artificial membrane, t is the permeation time, whilst d_a_ and d_eq_ are the absorbance measured in the acceptor well and the theoretical equilibrium absorbance value, respectively. After the PAMPA-BBB test for all compounds was completed, the integrity of the lipid membrane was evaluated based on the transport of Lucifer Yellow (Sigma-Aldrich)—a fluorescent molecule with very limited membrane permeability rejected by a uniform and integral lipid membrane performed according to the Millipore protocol lit. n. PC1545EN00 (https://www.sigmaaldrich.com/technical-documents/protocols/biology/membrane-integrity-test-for-lipid-pampa-artificial-membranes.html).

## Results

### Development of a combinatorial library of cyclic peptides (CYCLIC) suitable for Yeast Two-Hybrid assays

To develop a Y2H-based screening strategy useful for the identification of cyclic peptides that physically interact with selected target proteins, a modified version of the *dnaE* split intein-ecoding gene (SspIntein) from *Synechocystis* spp. PCC6803 (Barreto and Geyer, 2014) was cloned downstream from the GAL4-Activation Domain (GAL4-AD), into the pGADT7-KanMX vector (Supplementary Figure S1). The combinatorial peptide-encoding sequences, comprising eight consecutive NNK degenerate codons (8xNNK), were inserted between the Intein_C_ (C-terminal) and Intein_N_ (N-terminal) domains, through *in yeast* homologous recombination (Supplementary Figure S1). After transformation, 5 x 10^6^ independent clones were obtained and harvested. The resulting library, named CYCLIC (combinatorial library of cyclic peptides), enables the cyclization of peptides by Intein_N_ excision and lactone bond formation between the hydroxyl group of a serine side chain (named Ser_1_) and the C-terminus of the amino acid encoded by the 8^th^ NNK codon (Barreto and Geyer, 2014).

The effectiveness of our library as a platform for the identification of target protein-interacting cyclic peptides was tested, using the GAL4-DNA Binding Domain (GAL4-DBD) as a bait in the Y2H screen. To this purpose, the yeast strain AH109 (*MATa*), harboring the CYCLIC library, was co-cultivated with the strain Y187 (*MATα*), expressing the GAL4-DBD bait protein, to favor the formation of diploid cells essential for the screening (Figure 1A). By sequencing the colonies grown on selective media, 99 cyclic peptides physically interacting with the GAL4-DBD were identified and named G4CPs (GAL4-DBD-interacting cyclic peptides) (Supplementary Table S2). A randomly selected subset of peptides was assayed for successful intein-mediated splicing and cyclization by immunoblot using an anti-GAL4-AD antibody (Figure 1B). Two protein bands, migrating at 38 and 23 kDa, represent the uncyclized and cyclic forms of the peptide (Barreto et al., 2009), respectively, that coexist together with cyclization intermediates in yeast cells. Furthermore, the strength of the interaction with the GAL4-DBD was assayed by plating diploid cells (expressing the subset of G4CPs) on selective medium supplemented with 2.5 mM 3-AT (Figure 1C). Three peptides, named G4CP2, 36 and 64, were selected for further analyses as they showed different levels of interaction strength with the bait (Figure 1C). The G4CPs identified (Figure 1B) were expressed in a haploid AH109 strain in the absence of the GAL4-DBD. The yeast colonies were unable to grow on selective media, thus excluding that they could trigger the transcription of the report genes in the absence of GAL4-BD (Supplementary Figure S3).

### GST-G4CP2 reduces the ability of the endogenous yeast GAL4 transcription factor to activate transcription

**FIGURE 2:**
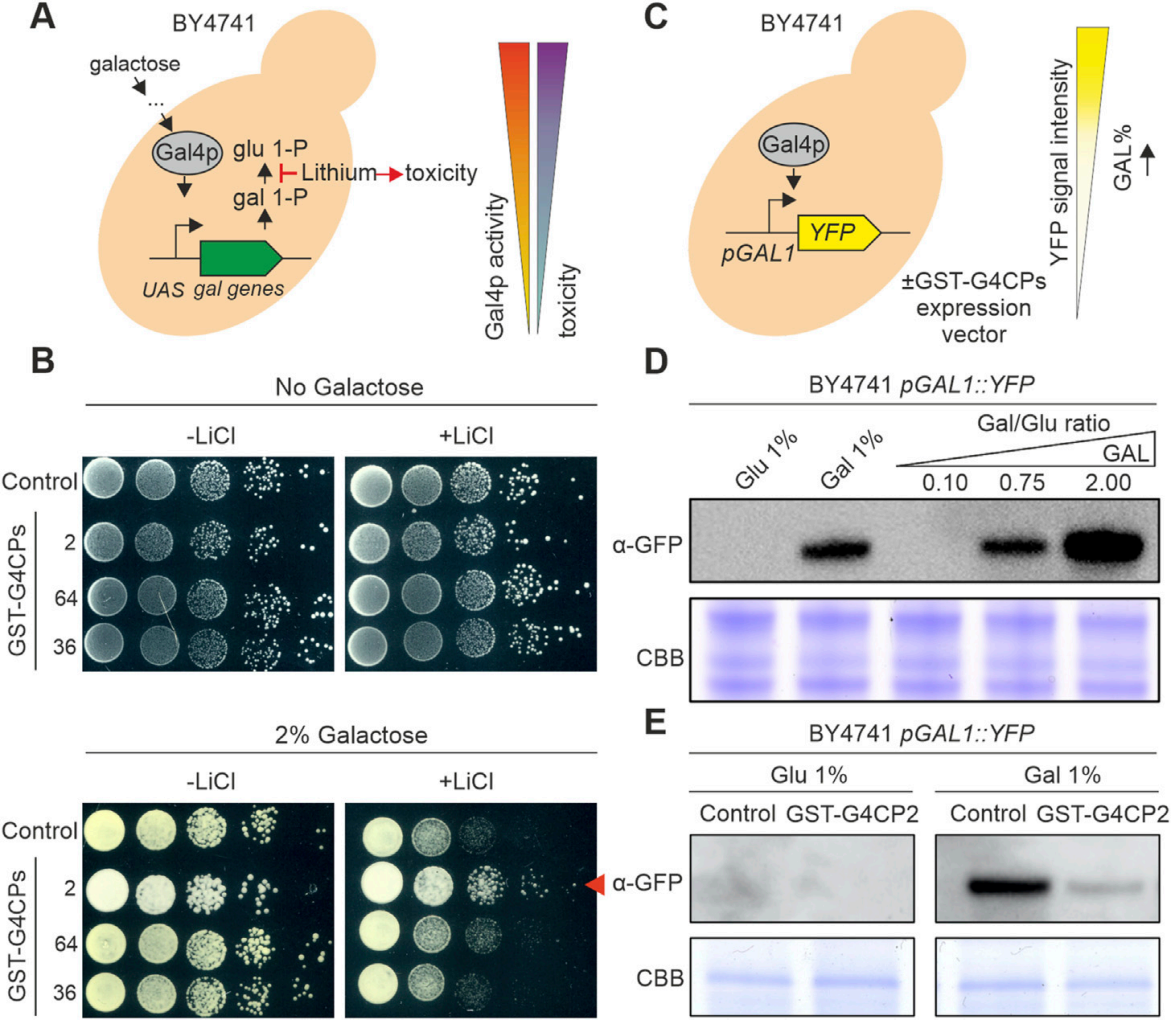
GST-G4CP2 reduces GAL4 transcriptional activity. **(A)** Galactose metabolism. Galactose entry into yeast cells activates the GAL4 transcription factor which, in turn, induces the transcription of galactose metabolic genes. In this context, lithium can block the conversion of galactose 1-phosphate (gal 1-P) to glucose 1-phosphate (glu 1-P), leading to cell toxicity and cell death in yeast. Inhibition of GAL4 activity in these conditions will allow higher growth rate by bypassing lithium toxicity. UAS, Upstream Activator Sequence. **(B)** Lithium toxicity assay. BY4741 yeast cells expressing, or not, different GAL4-interacting cyclic peptides (G4CPs) were grown on media supplemented with either 0% or 2% galactose, in the absence or presence of lithium. Relative growth of serial dilutions of each strain was monitored over a few days. The red arrowhead indicates the increased growth of GST-G4CP2-expressing strain on 2% galactose + lithium-containing medium. **(C)** BY4741 pGAL1:YFP reporter strain allows the monitoring of GAL4 activity *in vivo* and enables to assess the effects of GST-G4CP2 inhibitory activity. **(D)** Reporter response to different sugars and sugar ratio in the BY4741 strain, monitored by immunoblot analysis using a GFP specific primary antibody. **(E)** Immunoblot analysis using a GFP specific primary antibody performed on total proteins extracted from BY4741 pGAL1:YFP yeast cells expressing GST-G4CP2 or control plasmids, grown on either glucose or galactose supplemented media. C.B.B., Coomassie Brilliant Blue stained SDS-PAGE, used as loading control.

The addition of lithium to the yeast growth medium triggers yeast cell death when galactose is the main carbon source, because of the inhibition of phosphoglucomutase, a key enzyme in galactose metabolism (Figure 2A) (Masuda et al., 2008). Based on this, reduced activity of GAL4, under selective conditions (presence of galactose and lithium), should lead to increased yeast cell growth. A cytotoxicity assay was set up to evaluate the ability of selected G4CPs to interfere with GAL4 transcriptional activity. The prey vectors, expressing G4CP2, 64 and 36 were engineered by replacing the GAL4-AD with a GST tag (GST-G4CP2, GST-G4CP64 and GST-G4CP36) (Supplementary Figure S4) and introduced into the BY4741 yeast strain. This strain can grow on galactose-containing medium, since it carries the entire set of genes encoding the enzymes required for galactose metabolism, including the GAL4 transcription factor. BY4741 transformants were grown on YSD medium supplemented with either 0% or 2% galactose and in the absence or presence of 40 mM LiCl (Figure 2B). Yeast colonies, expressing either GST-G4CP64 or GST-G4CP36 and plated on medium with galactose and lithium, showed an evident delay in growth, whilst yeast colonies carrying the GST-G4CP2 construct grew vigorously on the same medium (Figure 2B).

This supports the notion that the binding of G4CP2 to GAL4 might interfere with GAL4 transcriptional activity and, therefore, reduces the accumulation of galactose-1-phosphate and other metabolic intermediates that are cytotoxic for yeast cells.

To further support this evidence, we introduced the *pGAL1*:YFP reporter vector into the BY4741 strain (Figure 2C), where *YFP* expression is controlled by the binding of GAL4 to the *GAL1* promoter (Escalante-Chong et al., 2015; Ricci-Tam et al., 2021). The BY4741 yeast strain responded quickly to the addition of 1% galactose to the growth medium and to different galactose/glucose (Gal/Glu) ratios (from 0.10 to 2.00), as shown by the large accumulation of YFP protein, monitored by immunoblot using a GFP-specific antibody (Figure 2D). The same strain was also transformed with either GST-G4CP2 or an empty plasmid (pGADT7-KanMX-GST-SspIntein) and the accumulation of YFP was monitored by immunoblot analysis. Interestingly, while the control and GST-G4CP2 expressing strains did not show any YFP accumulation in presence of 1% glucose, the activation of the reporter gene was strongly attenuated in the presence of GST-G4CP2 under inducing conditions (1% galactose), confirming the interference of GST-G4CP2 with GAL4 transcriptional activity.

GST-G4CP2 should also be able to affect the expression of GAL4 target genes. To verify this assumption, we performed real time (RT)-qPCR analysis, comparing the expression of the *GAL1* (Lohr and Hopper, 1985) and *GAL2* (Huibregtse et al., 1993) genes between control (pGADT7-KanMX-GST-SspIntein) and GST-G4CP2-expressing strains (in the BY4741-*pGAL1::YFP* genetic background). As highlighted in Supplementary Figure S5, significant lower *GAL1* and *GAL2* expression levels were detected for the GST-G4CP2-expressing strain, compared to the control, while no differences were observed using glucose as carbon and energy source.

To confirm the interaction between GST-G4CP2 and GAL4-DBD, a pull-down interaction assay was also performed. The physical protein-protein interaction is shown in Supplementary Figure S6.

### GST-G4CP2 reduces GAL4 activity over a wide range of gal/glu ratios

**FIGURE 3:**
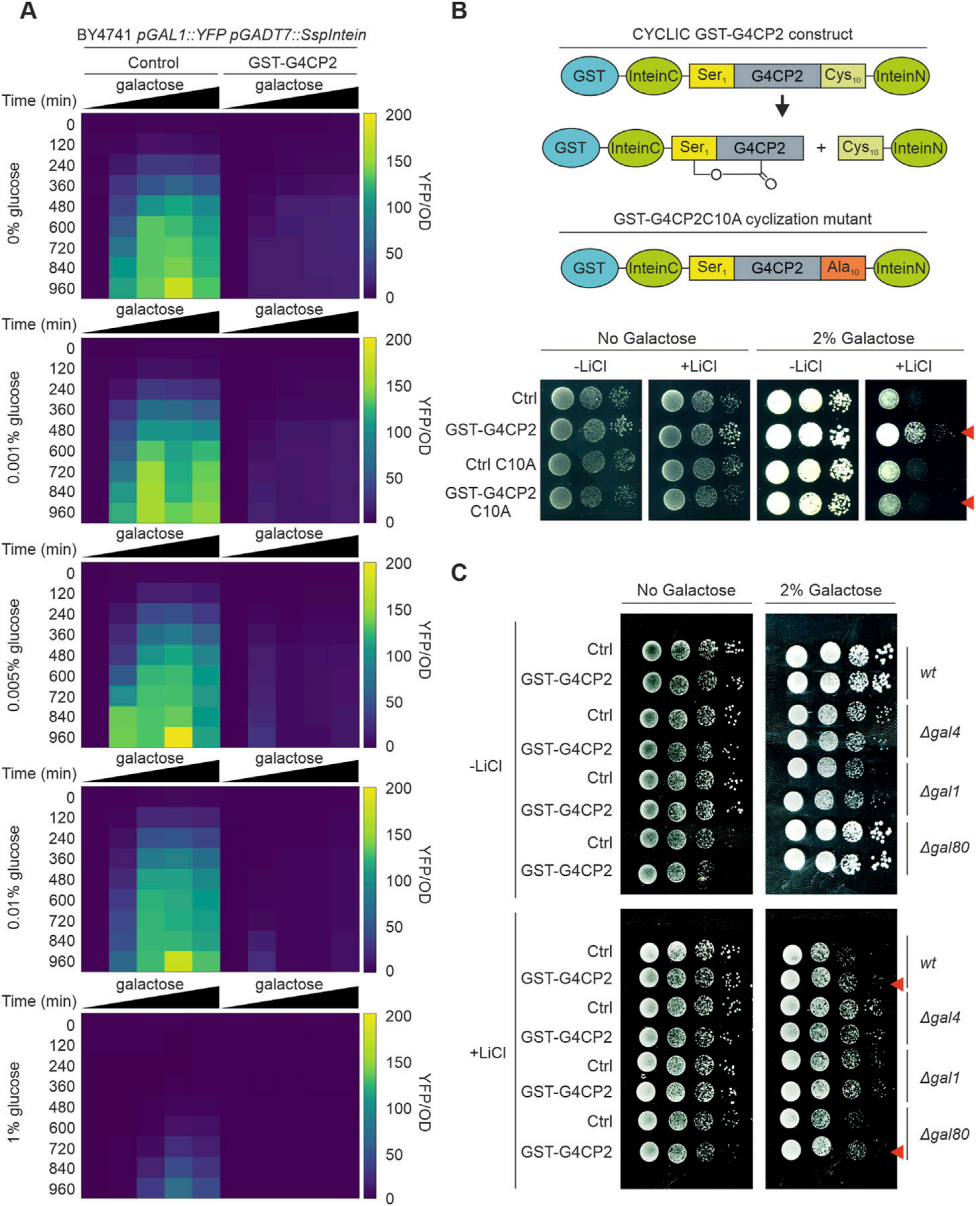
GST-G4CP2-mediated inhibition of GAL4 occurs over a wide range of sugar concentrations and GST-G4CP2 cyclization is required for activity. **(A)** High-throughput YFP fluorescence signal measurement of BY4741 *pGAL1::YFP* yeast cells expressing, or not, GST-G4CP2 and grown under different galactose/glucose ratios in 96-well plates over a timeframe of 15.5 h. Fluorescence read-out is given as an heatmap and expressed as YFP signal/OD_600_ (for raw data see Supplementary Table S4). **(B)** Mutation of Cysteine 10 to Alanine (C10A) in the peptide scaffold prevents peptide cyclization, as previously reported by Barreto et al. (2009). Lithium toxicity assay performed in yeast cells expressing either GST-G4CP2 or the mutant version GST-G4CP2-C10A, with their respective controls (empty vectors), highlight increased tolerance of the GST-G4CP2-expressing strain to lithium with respect to the GST-G4CP2-C10A-expressing strain. **(C)** Lithium toxicity assay in *gal* mutant backgrounds (*Δgal1*, *Δgal4* and *Δgal80*) expressing, or not, GST-G4CP2, to assess the impact and specificity of GST-G4CP2 on GAL4 activity upon perturbation of galactose metabolism. Red arrows indicate genetic background where GST-G4CP2 induced higher lithium tolerance.

To further characterize the effects of GST-G4CP2 on the target protein, we measured the activity of GAL4 in BY4741 yeast cells, carrying the *pGAL1::YFP* reporter gene, with or without GST-G4CP2, as an average of YFP fluorescence signal intensity over yeast cell count (YFP/OD_600_). To this purpose, we used 96-well plates containing media with different galactose/glucose ratios (Figure 3A; Supplementary Table S4), as galactose sensing in yeast is influenced by the galactose/glucose ratio in the medium, rather than on galactose concentration alone (Escalante-Chong et al., 2015). As expected, the YFP fluorescence signal of the control strain increased in response to increasing relative galactose concentrations, while almost no YFP signal was detected from the strain expressing GST-G4CP2, further confirming the interference of GST-G4CP2 on GAL4 activity over a wide range of Gal/Glu ratios. As highlighted in Supplementary Figure S4, GST-G4CP2 exists in both un-cyclized (unspliced intein) and cyclized (spliced intein) forms in yeast cells. To verify that only the cyclic version of GST-G4CP2 interferes with GAL4 activity, a split-intein variant, where cysteine in position 10 is replaced with an alanine (C10A) to prevent cyclization, was produced (Barreto et al., 2009) (Figure 3B). The lithium toxicity assay was subsequently repeated, revealing that, while the GST-G4CP2-expressing strain retains an increased capacity for growth on selective media (2% galactose and 40 mM lithium chloride), the GST-G4CP2-C10A mutant version is unable to induce any positive effect on yeast growth, with the latter being comparable to the control. This result demonstrates the relevance of G4CP2 cyclization to hinder GAL4 transcriptional activity. To strengthen the assumption that only the cyclic version of G4CP2 is responsible for the observed phenotypes, we generated a G4CP2 variant (linear G4CP2), where a stop codon is inserted between the G4CP2 sequence and the Intein_N_ element (Supplementary Figure S7A). This modification prevents Intein_N_ expression and the consequent peptide cyclization, leaving a linear G4CP2 sequence attached to the GAL4AD-InteinC fusion protein (Barreto et al., 2009). Western blot analysis, confirmed the absence of Intein_N_ expression and circularization of the peptide (Supplementary Figure S7B). Consistently, the yeast strains expressing the linear G4CP2 failed to grow on media lacking tryptophan, leucine, adenine and/or histidine since it was unable to interact with GAL4-DBD and activate the reporter genes *ADE2* and *HIS3* (Supplementary Figure S7C).

### GST-G4CP2 is specific for the GAL4 transcription factor

GAL1 and GAL80 are positive and negative regulators of the GAL4 TF, respectively (Supplementary Figure S8), and their roles in the regulation of galactose metabolism in yeast have been extensively studied (Harrison et al., 2022). To further characterize the effects of GST-G4CP2 on the endogenous galactose metabolic pathway, control and GST-G4CP2-expressing plasmids were introduced into different *gal* mutants (BY4741 genetic background), such as *Δgal4*, *Δgal1* and *Δgal80*, and their growth on lithium-containing medium was evaluated (Figure 3C). When grown on medium with 2% galactose and lithium, increased growth of GST-G4CP2-expressing BY4741 (wt) and *Δgal80* strains, where GAL4 is normally functioning or over-activated, respectively, was observed, while no differences in growth were detectable between *Δgal4* and *Δgal1* strains, since GAL4 is absent or strongly repressed in these genetic backgrounds, supporting the specificity of GST-G4CP2 for the GAL4 transcription factor.

### Unconstrained G4CP2 maintains its ability to inhibit the GAL4 TF

**FIGURE 4:**
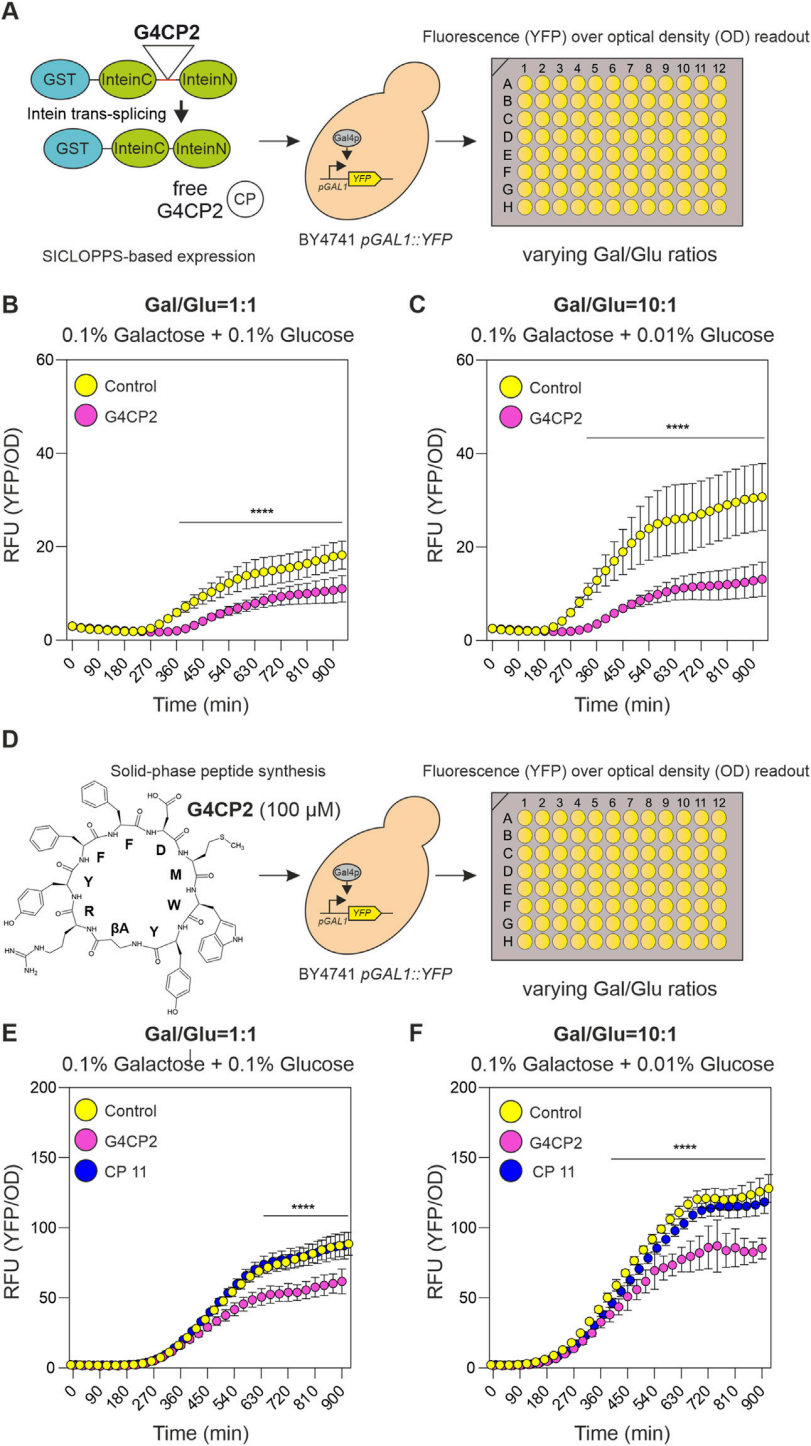
Unconstrained G4CP2 retains its GAL4 inhibitory activity. **(A)** On the left, a schematic representation showing how GST-SICLOPPS works. After intein splicing, the cyclic peptide (CP) is excised from the reconstituted intein (Intein_C_ + Intein_N_) and released intracellularly without any additional scaffold. Cells expressing control (GST-SICLOPPS) or G4CP2 (GST-SICLOPPS-G4CP2) were grown in 96-well plates under two different galactose/glucose ratios, non-inducing conditions (1:1) and inducing conditions (10:1), shown in panel **(B and C)**, respectively. Fluorescence signals were recorded over a timeframe of 930 min, and reported in the graphs as YFP signal intensity/OD_600_. **(D)** Chemically synthesized G4CP2 (shown on the left) and CP11 (not shown) were applied to BY4741 *pGAL1::YFP* yeast strain at 100 µM. Cells were treated with G4CP2, CP11 or mock (Control) and grown in 96-well plates and replicating the conditions described for the experiment in the panel **A**. Fluorescence read-out is expressed as YFP signal intensity/OD_600_ (single graphs for YFP and OD_600_ are reported in single YFP and OD graphs are Supplementary Figure S11). Statistical significance was determined using Two-way ANOVA: *p < 0.05, **p < 0.01, ***p < 0.001, ****p < 0.001. Raw data (*n = 16*) are shown in Supplementary Table S5.

While GST-G4CP2 can interfere with GAL4 activity when expressed intracellularly and fused to a scaffold such as GST-SspIntein, the GAL4-interfering activity of free G4CP2 when it is provided alone without any scaffold fused to it remains to be investigated. To verify whether the unconstrained G4CP2 can interfere with the GAL4 TF, we used two different strategies. In the first case, we mutated the intein to the one used in the SICLOPPS system (A36N), which releases a scaffold-free cyclic peptide intracellularly after self-splicing. Alternatively, the effect of chemically-synthesized, exogenously-added cyclic peptide was evaluated in yeast cell culture. Unfortunately, the ester bond present in the lactone structure of G4CP2 is known to be subject to hydrolysis, possibly resulting in the linear carboxylate derivative. To avoid G4CP2 linearization, the G4CP2 peptide (βA-RYFFDMWY) containing a βAlanine (βA) instead of Ser_1_ (see Figures 1B, 4D) was produced. This bioisosteric replacement allowed to introduce the lactam bond that is more stable toward hydrolysis than the lactone one, without altering the number of atoms of the cyclic peptide backbone.

To perform βA-RYFFDMWY synthesis, the chlorotrityl resin was chosen as a solid support as it allows the full protected peptides to be cleaved from the resin. Head-to-tail cyclization was then performed in-solution in pseudo-diluted conditions (0.2 mM in DMF), using PyBOP and HOBt as coupling reagents and DIPEA as the base. After cyclization, the side chain protecting groups were removed and the crude peptide was purified on RP-HPLC affording the pure G4CP2 cyclic peptide.

SICLOPPS-based control (pGADT7-KanMX-GST-SICLOPPS) and G4CP2 (pGADT7-KanMX-GST-SICLOPPS-G4CP2) constructs were introduced in the BY4741 *pGAL1::YFP* strain, and the activity of the biosensor in the two strains, grown in 96-well plates under 1.00 and 10.00 Gal/Glu ratios, was recorded again as YFP/OD_600_ signal over a time-frame of 15.5 h (Figure 4A). As for the lariat-generating construct (Figure 3A), expression of free G4CP2 induced a significant decrease in reporter signal, indicating that it retained the ability to inhibit GAL4 downstream activity (Figures 4B,C; Supplementary Table S5, Supplementary Figure S9).

To test the effects of exogenous administration of chemically synthetized G4CP2 on *pGAL1::YFP* biosensor, a random-selected control peptide (CP11) was used. Absence of toxicity for yeast cells was demonstrated for both CPs (Supplementary Figure S10). G4CP2 and CP11 were used at 100 µM to treat BY4741 *pGAL1::YFP* yeast cells grown in 96-well plates under 1.00 and 10.00 Gal/Glu ratios. YFP/OD_600_ values were again recorded for 15.5 h (Figure 4D).

As shown in Figures 4E, F (see also Supplementary Table S5), reporter activation occurs at both 1.00 and 10.00 Ga l/Glu ratios, while being stronger, consistently, when the concentration of glucose was lowered by ten times (Figure 4F). Interestingly, non-treated and CP11 treated cells showed a continuous increase in YFP fluorescence signal, while this effect was strongly attenuated in G4CP2-treated cells (Figures 4E, F). Clearly, this difference cannot be attributed to different growth rates, rather than to different activation of the YFP reporter, as shown by the single YFP and OD graphs (Supplementary Figure S11), demonstrating that the unconstrained G4CP2 can interfere with GAL4 activity.

Our bioisosteric replacement approach is commonly used in medicinal chemistry to obtain clinically effective compounds (Patani and LaVoie, 1996), although differences between lactone and lactam bonds are reported in literature (Kerns et al., 2004). Specifically, the chemically-synthetized lactam version of G4CP2 is able to mimic the biological activity of the genetically expressed lactone counterpart (GST-G4CP2), indicating that no major alterations on G4CP2 activity are introduced upon lactone/lactam bond replacement.

The successful exogenous application of G4CP2 suggests that the cyclic peptide is able to move through the yeast cell wall and plasma membrane. To study G4CP2 cell permeation, the *in vitro* PAMPA-BBB assay was carried out to determine the value of the passive membrane permeability P_e,exp_ for G4CP2 and three further cyclic peptides, namely CSA, CSD, and EnB. According to the adopted passive permeability classification scheme (Di et al., 2003; Campora et al., 2021), the P_e,exp_ value obtained for G4CP2 (3.3 ± 0.4 ×10^−6^ cm/s) allowed this cyclic peptide to be classified as a moderate-to-good membrane-permeable compound, in particular when compared to CSA (P_e,exp_ = 2.1 ± 0.4 ×10^−6^ cm/s), CSD (P_e,exp_ = 0.93 ± 0.5 ×10^−6^ cm/s), and to EnB (P_e,exp_ = 8.3 ± 0.7 ×10^−6^ cm/s), for which methylation of all N atoms involved in the peptide bonds is known to positively modulate membrane permeability (Dougherty et al., 2019). PAMPA-BBB assay validation was finally performed by determining the experimental permeabilities for 24 control compounds of known passive permeability values (P_e,lit_), yielding very good overall data agreement (Supplementary Table S3). The overall satisfactory membrane permeability of G4CP2 is further highlighted by the fact that PAMPA-BBB uses a blood-brain barrier, which is more difficult to cross than other barriers used in PAMPA assays.

## Discussion

In this work, we adapted an 8xNNK combinatorial library of cyclic peptides to the GAL4-based Yeast Two-Hybrid assay, enabling the identification of cyclic peptides that can interact with a target protein (bait). Here, we show that CYCLIC can be used to identify CPs (Supplementary Table S2) with binding affinity and inhibitory activity toward the full-length GAL4 TF, as shown for G4CP2. This peptide represents a useful active molecule to be used in any biotechnological approach that relies on the yeast GAL4 TF, as it exerts its inhibitory activity when both heterologously expressed or exogenously applied.

Beyond CYCLIC, the implementation of split-inteins in A TF-dependent selection system was already reported in literature. Head-to-tail cyclic peptides obtained *via* intein splicing have been identified for their ability to avoid cell death in human (Kinsella et al., 2002) and yeast (Kritzer et al., 2009) cells by interfering with biological processes upstream or downstream, respectively. However, in these studies, the authors did not identify the precise molecular targets of the identified CPs, thus the molecular mechanisms at the basis of their activities remain elusive. Recently, King and co-workers (2021b) developed a library of modified peptides, RiPPs (ribosomally synthesized and post-translationally modified peptide). The library relies on two fusion proteins containing the RiPP and the bait. Their interaction brings together two-halves of a split intein releasing a σ factor that recruits the RNA polymerase to promote transcription of a marker gene (King et al., 2021). This tool led to the identification of a SARS-CoV-2 Spike receptor binding domain (RBD) interacting peptide (AMK-1057). Interestingly, the interaction was not mapped on a known therapeutic binding region of the RBD protein, indicating that this library can be used for proteins that lack obvious “druggable” pockets and, as the assay is performed by screening the library on the entire protein surface, raising the possibility of developing’s peptides suitable for diagnostics and PROTACs (PROteolysis Targeting Chimeras).

The concept of screening a peptide library against the whole protein surface was anticipated by Barreto and collaborators (2009) that developed a LexA-based yeast two-hybrid assay for the identification of bait-binding “lariat” peptides. This strategy proved to be useful for the identification of inhibitors of LexA auto-proteolysis (Barreto et al., 2009) and of the kinase ABL1 (Bharathikumar et al., 2013), involved in bacterial SOS response and chronic myelogenous leukaemia. In this context, CYCLIC is implemented in a GAL4-based system, bringing together the advantages related to one of the more widely adopted systems to study PPIs in molecular biology (Paiano et al., 2019) and the possibility to “blindly” assay the entire target molecular surface, particularly useful when “druggable” pockets or relevant PPIs are unknown, or inhibition/interference is not the desired research goal.

Additionally, CYCLIC is the first constructed and screened “lariat” peptide library having the structure Ser_1_-(X)_n_-Cys_10_ (X = any amino acid), discussed by Barreto and Geyer (2014), but never tested experimentally. All the other Y2H-compatible “lariat” peptide libraries reported in the literature (Barreto et al., 2009; Bharathikumar et al., 2013) displayed a peptide structure (Ser_1_-(X)_n_EY-Cys_10_) having a constitutive interaction bias derived by the presence of C-terminal E and Y residues in all the peptide sequences.

*Saccharomyces cerevisiae* GAL4 has been, indeed, a pioneer protein for synthetic biology applications, being at the basis of the well-known GAL4-based Yeast Two-Hybrid strategy. Moreover, the GAL4-upstream activating sequence (UAS) is a tool routinely used for targeting gene expression that has shed light molecular networks modulating tissue and organ differentiation in *Drosophila*, mouse, zebrafish, *Arabidopsis thaliana* and many other model organisms (Jiang et al., 2015; Slomovic et al., 2015; Ryo et al., 2017; Andres et al., 2019; Iacopino et al., 2019; Zhao et al., 2021).

Moreover, the list of G4CPs provided in this work, represents the “background noise” of our CYCLIC GAL4-based YH2 strategy, i.e. such a set of interacting peptides can be subtracted from the list of cyclic peptides identified by using any bait of choice fused to GAL4-DBD, reducing the downstream work of CP-bait interaction validation.

Noteworthy, by screening CYCLIC toward GAL4-DBD we were able to identify a cyclic peptide displaying inhibitory activity toward GAL4, even though we relied on a GAL4-based Y2H method, where GAL4-DBD is an essential module for the assay. As a matter of fact, a complete inhibition of GAL4BD would have compromised yeast growth on interaction-selective media and the consequent recovery of positive colonies. The fact that we were able to identify a bioactive peptide using GAL4-DBD as bait suggests that inhibitors can be found by screening CYCLIC with other targets.

Notably, no altered growth phenotype was observed in GST-G4CP2-expressing yeast cells, when grown on medium supplemented with galactose and in the absence of lithium, compared to their controls. In fact, even the *∆gal4* strain was still able to grow under these conditions (Figure 3C), suggesting unexplored players in the galactose metabolic pathway, in agreement with Masuda et al. (2008).

It should be highlighted that the peptide-bait protein interaction does not necessarily imply interference with target protein activity. As shown for G4CP2, the development of proper stand-alone tests to directly monitor bait protein activity after treatment with candidate cyclic peptides, such as luminescence/fluorescence-based reporter assays or enzymatic assays, is also needed for the discovery of active compounds. In this context, as shown in Figure 4, SICLOPPS is a good complementary assay to be used in combination with CYCLIC, providing a fast interaction-to-activity route for the identification of lead compounds in drug discovery studies, when *in vivo* functional/reporter assays are available.

Additionally, as shown in Supplementary Table S2, some GAL4 interacting peptides were identified several times during screening (sequence abundance). Consensus sequences could be recognized among all peptides identified using CYCLIC against the GAL4DBD as bait (Supplementary Figure S2 and Supplementary Table S2). However, abundance can only suggest good binding strength without implying any kind of activity towards the target protein. Furthermore, consensus sequences can be tricky to be interpreted since the molecular weight (size) of the target protein and library complexity must also be considered. The larger the molecular surface to be assayed, the higher the number of sequence motifs are identified (due to multiple binding sites). In addition, in libraries like CYCLIC (Barreto et al., 2009; Kritzer et al., 2009), not all possible amino acid combinations can be effectively screened. CYCLIC is constituted by 5 x 10^6^ yeast transformants, while peptides are codified by 32 possible NNK codons occupying 8 consecutive positions, thus 32^8^ = 1.1 x 10^12^ possible codon combinations are possible. Clearly, a low portion of the theoretical complexity can be effectively entrapped in the library. Therefore, the obtainment of robust consensus sequences is not guaranteed, especially when large protein surfaces are assayed. Probably, this analysis can provide useful information when performed on a sub-set of sequences having something in common, like the inhibition of enzymatic activity.

It is worth noting that peptides having been selected in Figures 1B, C, as stated above, were chosen randomly among G4CPs, without considering any consensus sequence analysis (Supplementary Figure S2, Supplementary Table S2) or their relative abundance in the screening (Supplementary Table S2), since these parameters do not provide information on their biological activities. In fact, we estimated that peptides capable of interfering with GAL4-DBD activity would be less represented with respect to those which are simply bait-interacting peptides (i.e., interaction without negative effect on GAL4-DBD), as their selection is unfavored by the choice to target an essential yeast two-hybrid module. Coherently, it is not surprising that none of the identified consensus sequences correctly describe G4CP2 amino acid composition, and that G4CP2 was identified just once in the screening.

Cyclic peptides have already been proven to be good candidates in drug discovery. In fact, peptides can be small, cyclization can improve their conformational stability, they are resistant to degradation and they can exhibit higher specificity for their target protein with respect to their linear counterparts. These features, together with their facile production using micro-organisms through fermentation, other than by chemical synthesis, makes them optimal candidates for the development of peptide-based drugs (Parachin et al., 2012; Bockus et al., 2013; Tapeinou et al., 2015; Yoshimi et al., 2018; Bucci et al., 2020; Cheng and Hua, 2020; Sohrabi et al., 2020; Kelly et al., 2021; Mejía-Pitta et al., 2021; Mendive-Tapia et al., 2021; Muttenthaler et al., 2021; Zhang and Chen, 2022). As shown in Figure 4, the unconstrained G4CP2 mirrored the molecular phenotype observed through expression of the GST-SspIntein fusion variant, confirming that this CP has inhibitory activity against GAL4 even when separated from the GST-SspIntein molecular scaffold. As indicated by the *in vitro* PAMPA-BBB assay, G4CP2 retains a moderate-to-good passive permeability, a very desirable feature for a cyclic peptide (Dougherty et al., 2019), especially when targeting intracellular proteins or PPIs. Peptide activity can be improved by increasing its permeability to the membrane and/or favoring nuclear localization, through conjugation with cell-penetrating peptides (CPPs) and nuclear signal peptides (SPs), respectively (Guidotti et al., 2017), or by improving its stability and resistance to degradation by micro-encapsulation (Jain, 2020), or by determining the optimal concentration for dose-response. Moreover, the heterologous expression of CPs in living organisms was already proposed as an alternative approach to avoid chemical synthesis and delivery optimization of synthetic peptides, as they can be produced in response to external stimuli and disease markers (Mistry and Tavassoli, 2017).

Our technology may also have direct applications in the field of microbial biotechnology and synthetic biology. In fact, it has been shown that inhibition of GAL4 orthologs in other yeast species, may increase fermentation capacity and yield (Jiang et al., 2015). Cyclic peptides can also be used in synthetic biology, as modules to create novel synthetic protein-protein interaction networks (Rosa et al., 2021) and for drugging proteins which usually are difficult to target, like transcription factors (Bushweller, 2019; Henley and Koehler, 2021), as in the case of G4CP2. Indeed, our screening procedure is based on mating the library-harboring strain with the bait-expressing strain. In this way, any target protein, from transcription factors to metabolic enzymes, from any organism, can be used as baits and tested *in vivo* over a few weeks, without the need of costly and time-consuming expression and purification steps for the recombinant bait proteins, typically required for *in vitro* assays. Nevertheless, it is worth mentioning that the selected bait protein (or a portion of it) may not be completely orthogonal to the screening host organism (yeast), due to its inherent ability to activate transcription, poor solubility and the presence of trans-membrane α-helices, thus preliminary *in vivo* auto-activation tests and *in silico* predictions of bait protein domains are mandatory. Finally, our *in vivo* screening procedure may allow the *a priori* elimination of peptides that are potentially toxic to eukaryotic cells, making it suitable for a plethora of applications in both basic and applied research fields.

## Data Availability

The data sets presented in this study can be found in online repository. Their accessions are in the Supplementary Material.
