# Supplementary material for: The cyclic peptide G4CP2 enables the modulation of galactose metabolism in yeast by interfering with GAL4 transcriptional activity
Source: Front Mol Biosci. 2023 Mar 1;10:1017757. doi: 10.3389/fmolb.2023.1017757 (PMC10014601; doi:10.3389/fmolb.2023.1017757)
Supplement: Supplementary file 1 [file DataSheet7.pdf]

Supplementary Figure S7

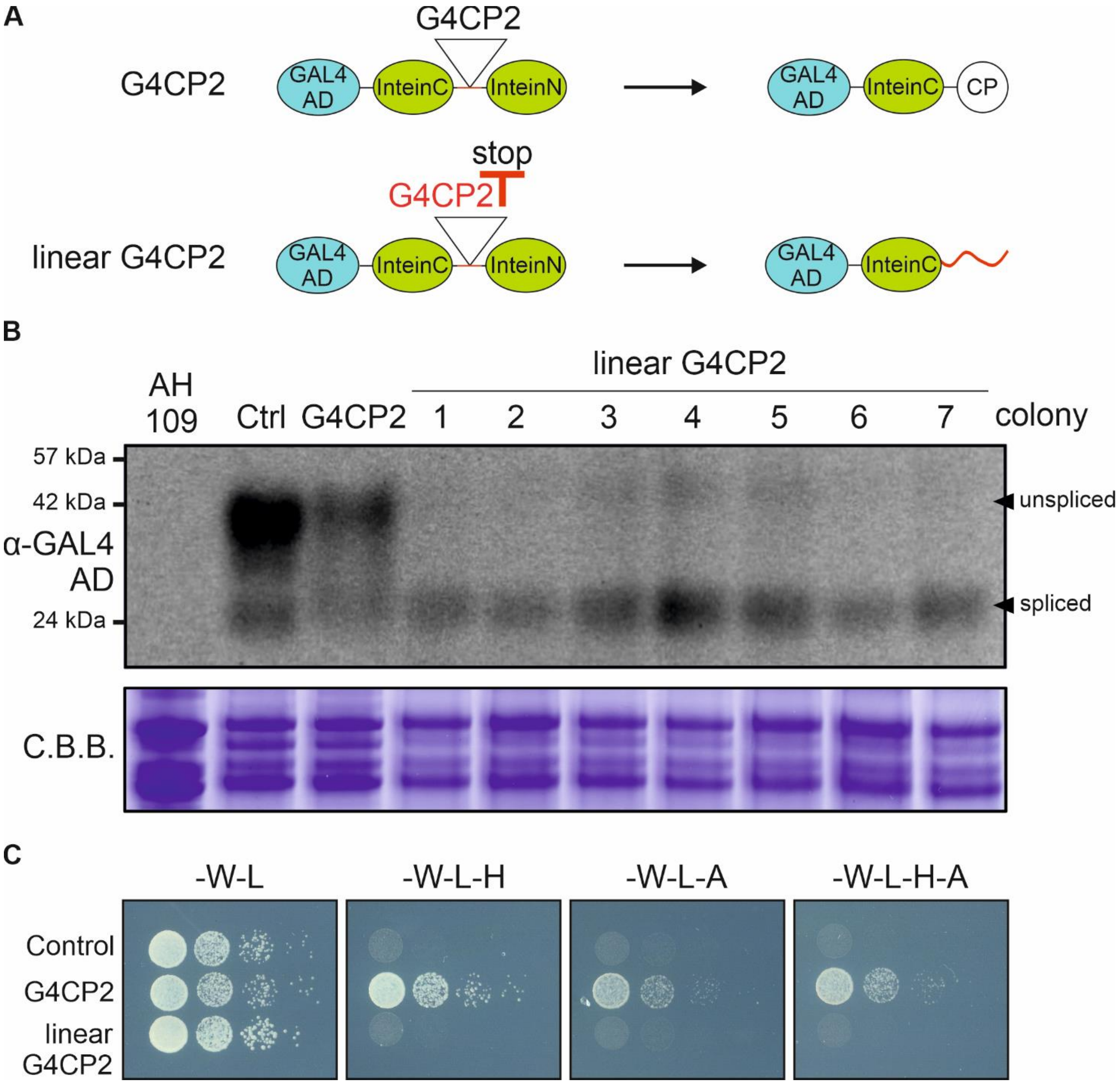

Supplementary Figure S7 – Linear G4CP2 does not interact with the GAL4DBD in Y2H assay.

A GAL4AD-G4CP2 genetic construct was modified by inserting a stop codon after G4CP2 sequence, thus preventing $Intein_N$ production and consequent splicing-mediated peptide cyclization (Barreto et al., 2009), producing a linearised version of G4CP2. **B** Western blot analysis using anti-GAL4AD antibody demonstrated that linear G4CP2 produce only the signal at a lower molecular weight corresponding to the spliced intein version in Control (GAL4AD-SspIntein) and G4CP2 (GAL4AD-G4CP2) lanes, thus proving the absence of InteinN production. **C** Y2H assay demonstrates the complete absence of growth of linear G4CP2-expressing strain on interaction-selective media (SD -W-L-H, SD -W-L-A and SD -W-L-H-A) when co-expressed with GAL4DBD.

This result testifies the relevance of G4CP2 cyclic form to produce a fruitful interaction with the GAL4DBD.
