# Supplementary material for: The cyclic peptide G4CP2 enables the modulation of galactose metabolism in yeast by interfering with GAL4 transcriptional activity
Source: Front Mol Biosci. 2023 Mar 1;10:1017757. doi: 10.3389/fmolb.2023.1017757 (PMC10014601; doi:10.3389/fmolb.2023.1017757)
Supplement: Supplementary file 2 [file DataSheet2.pdf]

Supplementary Figure S2

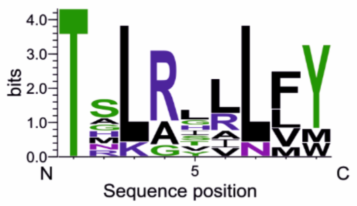

Consensus 1	**TXLRXXLXY**
Alignment	TMKRLLLF-
	-HLRYINFY
	-SLASRLVY
	-ALRTLLLW
	-SLGIALMY
	-RLAVVLFY
	-SLRLLLFY
	-NLRHLLVM
	-GLRGRLLY

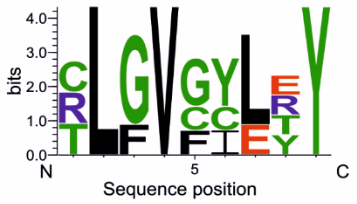

Consensus 2	**XLGVXXLXY**
Alignment	TLGVCCLY-
	CLGVFILR-
	RLGVGYLT-
	-LFVGYEEY

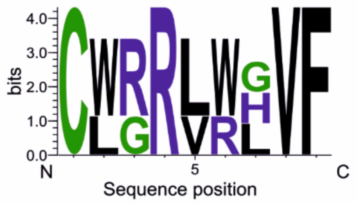

Consensus 3	**CWRRLWXVF**
Alignment	CWGRVWHV-
	-WRRLWLVF
	-LRRLRGVF

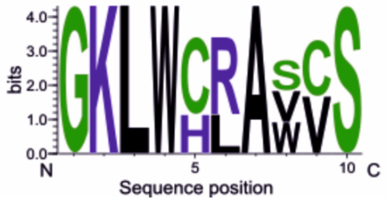

Consensus 4	**GKLWCRAXXS**
Alignment	GKLWCLAV--
	--LWHRAWCS
	--LWCRASVS

Consensus 5	**RCXSDWLTY**
Alignment	RCHMDMLT-
	-CWSDWLTY
	-CGSDWLTY

Consensus 6	**AMSLLXXR**
Alignment	CMSLMWLR
	AISMLSDR
	AMSLLAIR

Consensus 7	**VLRAXVXRQ**
Alignment	VLRSVVVR-
	VLRALVDL-
	-LRACVARQ

Consensus 8	**ILXRTXVX**
Alignment	ILMRCGVW
	ILVRTCVV
	LLNRTVVT

Consensus 9	**GILLXSLWF**
Alignment	GILLQSYW-
	-WLLFSLWF
	-ILLGALWF

Consensus 10	**XRLRXXXF**
Alignment	ARLREWRF
	IRLRRRGF

Consensus 11	**XXGGXVLX**
Alignment	RFGGKVLW
	SRGGPVLV

Consensus 12	**FRXRXRVXY**
Alignment	FRHRFRVR-
	-RVRSRVIY

Supplementary Figure S2– G4CPs consensus sequences.

The twelve identified clusters and resulting consensus sequences are reported in the tables, including the corresponding sequence alignment. Tables of consensus 1 to 4 are flanked by their sequence LOGOs obtained using WebLogo3 (Crooks et al., 2004). The complete consensus clustering result is reported in Supplementary Table S2.
