# Supplementary material for: The cyclic peptide G4CP2 enables the modulation of galactose metabolism in yeast by interfering with GAL4 transcriptional activity
Source: Front Mol Biosci. 2023 Mar 1;10:1017757. doi: 10.3389/fmolb.2023.1017757 (PMC10014601; doi:10.3389/fmolb.2023.1017757)
Supplement: Supplementary file 4 [file DataSheet4.pdf]

Supplementary Figure S4

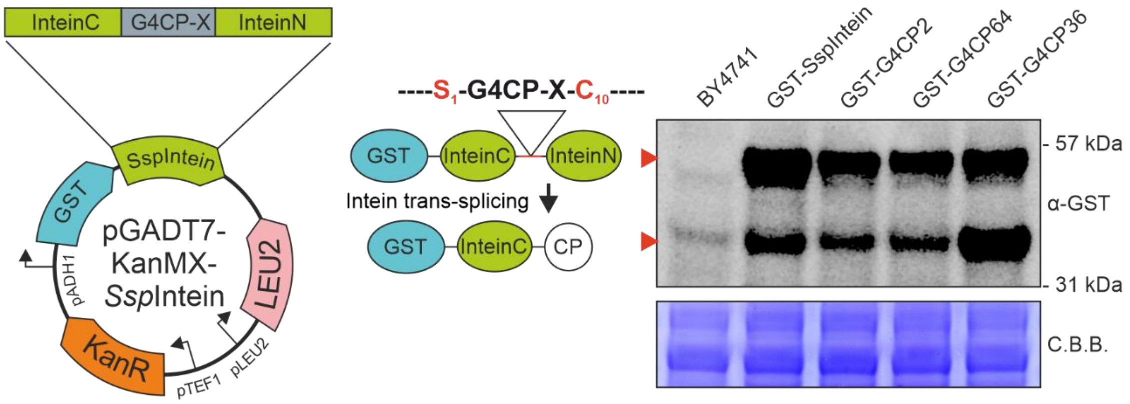

Supplementary Figure S4 – Intein-mediated cyclization of GST-G4CPs constructs.

The GAL4-AD-encoding cassette in the pGADT7-KanMX vector was replaced with a GST-tag. Protein extraction was performed from yeast cultures expressing either the three candidate GST-G4CPs (G4CP2, -64, -36) or the control (GST-empty) vector, and the intein-mediated splicing activity was evaluated through western blotting using an anti-GST antibody. As control, the untransformed yeast strain (BY4741 harbouring pGAL1:YFP reporter) was used. C.B.B., Coomassie Brilliant Blue stained SDS-PAGE, used as a loading control.
