# Supplementary material for: The cyclic peptide G4CP2 enables the modulation of galactose metabolism in yeast by interfering with GAL4 transcriptional activity
Source: Front Mol Biosci. 2023 Mar 1;10:1017757. doi: 10.3389/fmolb.2023.1017757 (PMC10014601; doi:10.3389/fmolb.2023.1017757)
Supplement: Supplementary file 5 [file DataSheet6.pdf]

Supplementary Figure S6

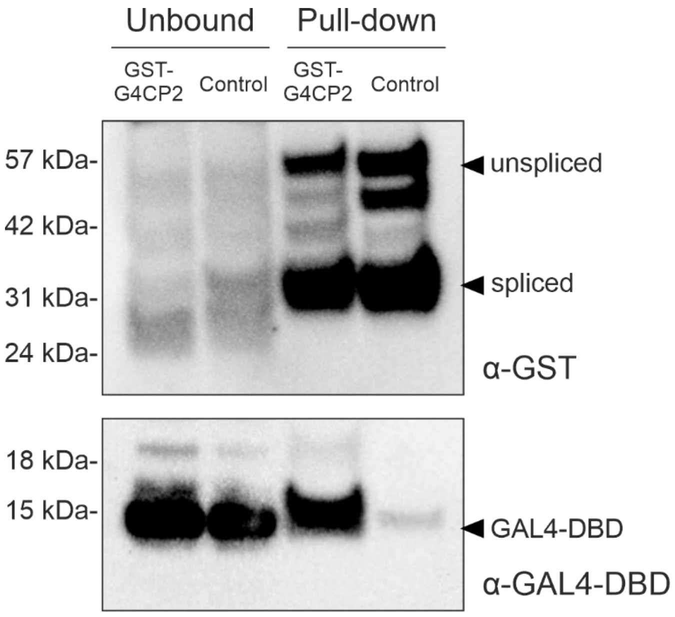

Supplementary Figure S6 – Pull-down assay displaying the physical interaction between GST-SspIntein-G4CP2 (GST-G4CP2) and GAL4-DBD.

GST-G4CP2 and GST-SspIntein (control) were expressed in yeast and isolated by glutathione sepharose resin. After a washing step, the glutathione resin was incubated with the cell lysate of E. coli expressing the GAL4-DBD. After formaldehyde crosslinking and extensive washes, coprecipitated proteins were eluted from the glutathione resin. A negligible GAL4-DBD signal was detected in the GST-SspIntein pull-down control, while GAL4-DBD was found to mainly accumulate in the GST-G4CP2 pull-down, proving the physical interaction with G4CP2. The pull-down supernatant was loaded as the Unbound fraction. Filters were then probed with GST and GAL4-DBD specific antibodies. The anti-GAL4-DBD primary antibody was purchased from Takara (Cat# 630403). Chemiluminescent signals corresponding to unspliced and spliced intein versions, and GAL4DBD are highlighted by black arrows.
