# Supplementary material for: The cyclic peptide G4CP2 enables the modulation of galactose metabolism in yeast by interfering with GAL4 transcriptional activity
Source: Front Mol Biosci. 2023 Mar 1;10:1017757. doi: 10.3389/fmolb.2023.1017757 (PMC10014601; doi:10.3389/fmolb.2023.1017757)
Supplement: Supplementary file 7 [file DataSheet9.PDF]

Supplementary Figure S9

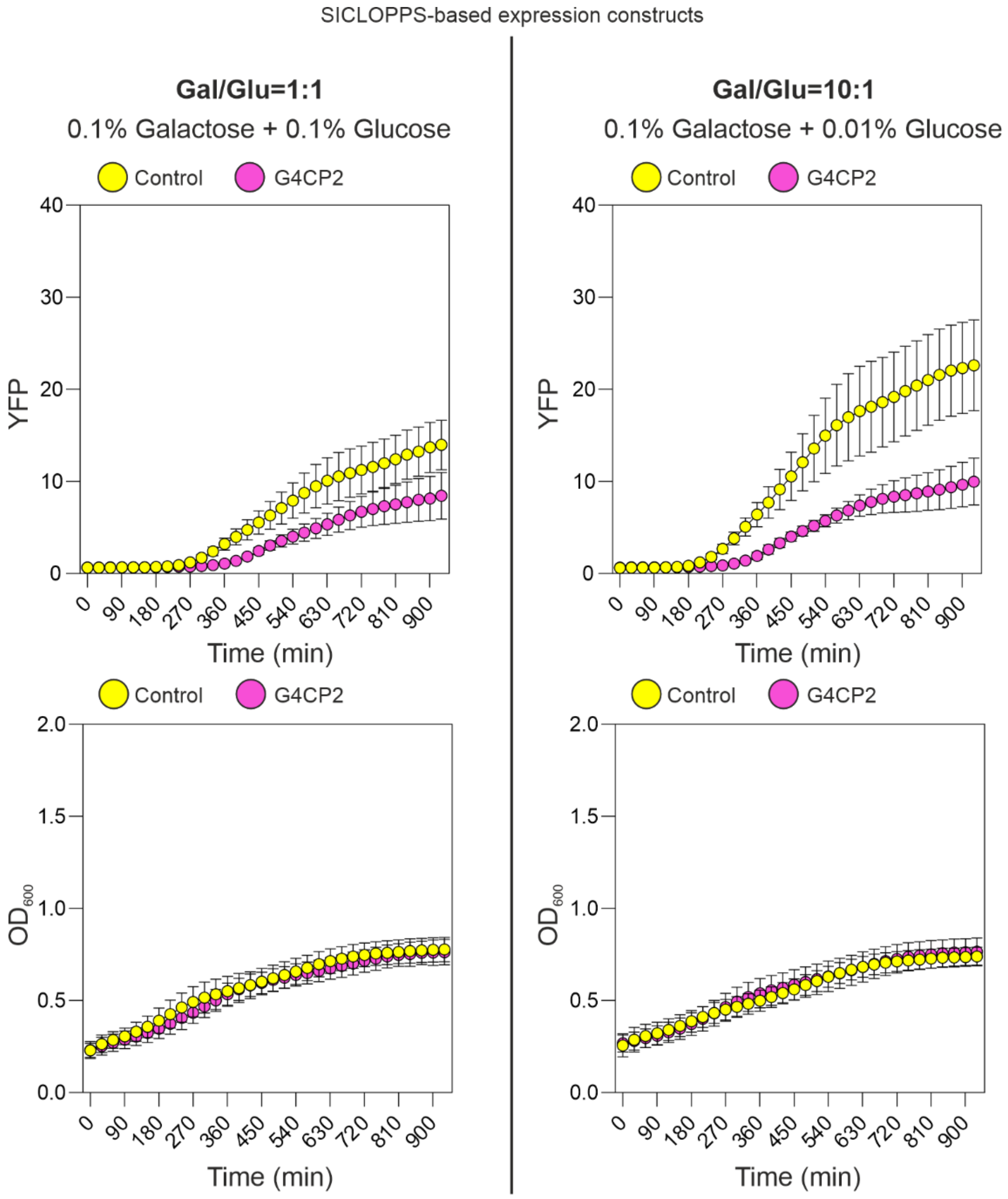

Supplementary Figure S9 – Single YFP and OD600 graphs for the experiment of Figure 4ABC.

Statistical significance (n=16) was determined using Two-way ANOVA: *p<0.001
