# Supplementary material for: The cyclic peptide G4CP2 enables the modulation of galactose metabolism in yeast by interfering with GAL4 transcriptional activity
Source: Front Mol Biosci. 2023 Mar 1;10:1017757. doi: 10.3389/fmolb.2023.1017757 (PMC10014601; doi:10.3389/fmolb.2023.1017757)
Supplement: Supplementary file 9 [file DataSheet3.pdf]

Supplementary Figure S3

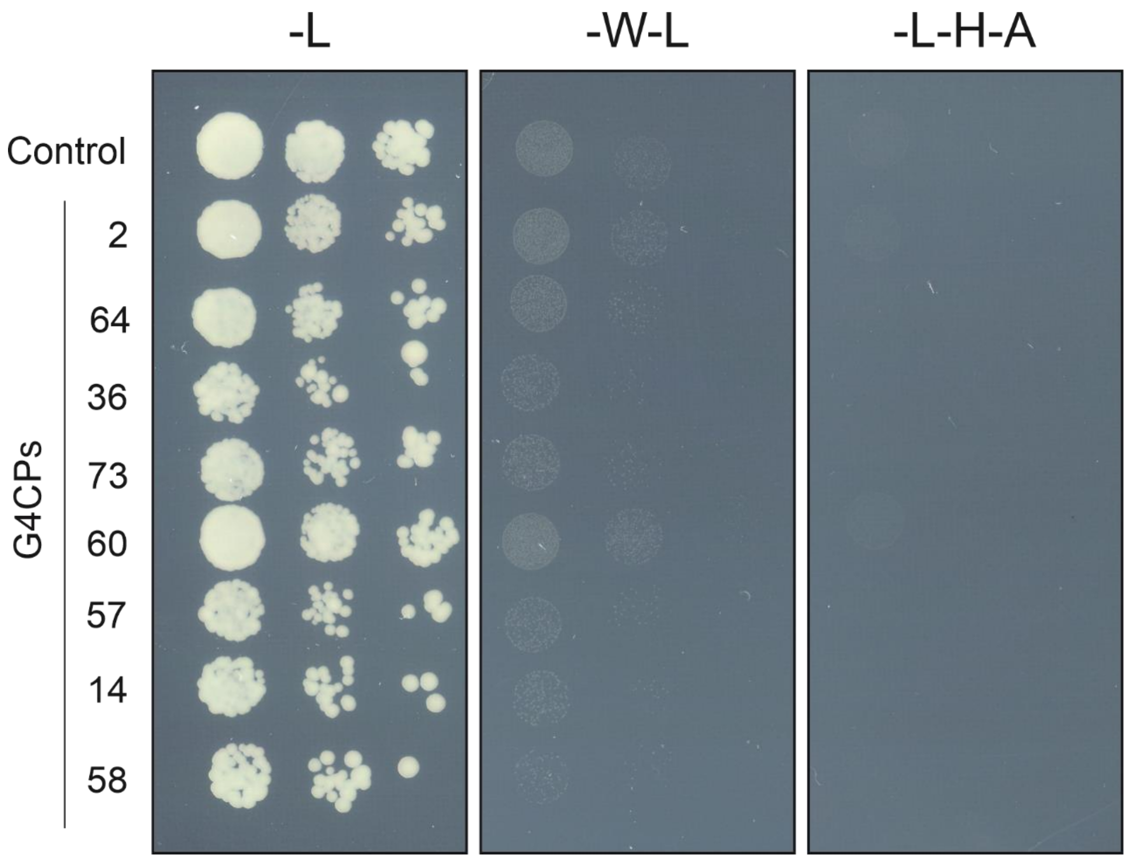

Supplementary Figure S3 – GAL4AD-G4CP fusions do not promote auto-activation of reporter genes in absence of GAL4DBD.

Haploid AH109 yeast cells expressing the same G4CPs reported in Figure 1C (without GAL4BD) were assayed on different selective media to verify whether they can promote the growth of yeast even in the absence of the bait protein. As a result, no yeast growth can be observed on -W-L and -L-H-A plates, demonstrating a complete absence of auto-activation.
