# Supplementary material for: The cyclic peptide G4CP2 enables the modulation of galactose metabolism in yeast by interfering with GAL4 transcriptional activity
Source: Front Mol Biosci. 2023 Mar 1;10:1017757. doi: 10.3389/fmolb.2023.1017757 (PMC10014601; doi:10.3389/fmolb.2023.1017757)
Supplement: Supplementary file 10 [file DataSheet1.PDF]

Supplementary Figure S1

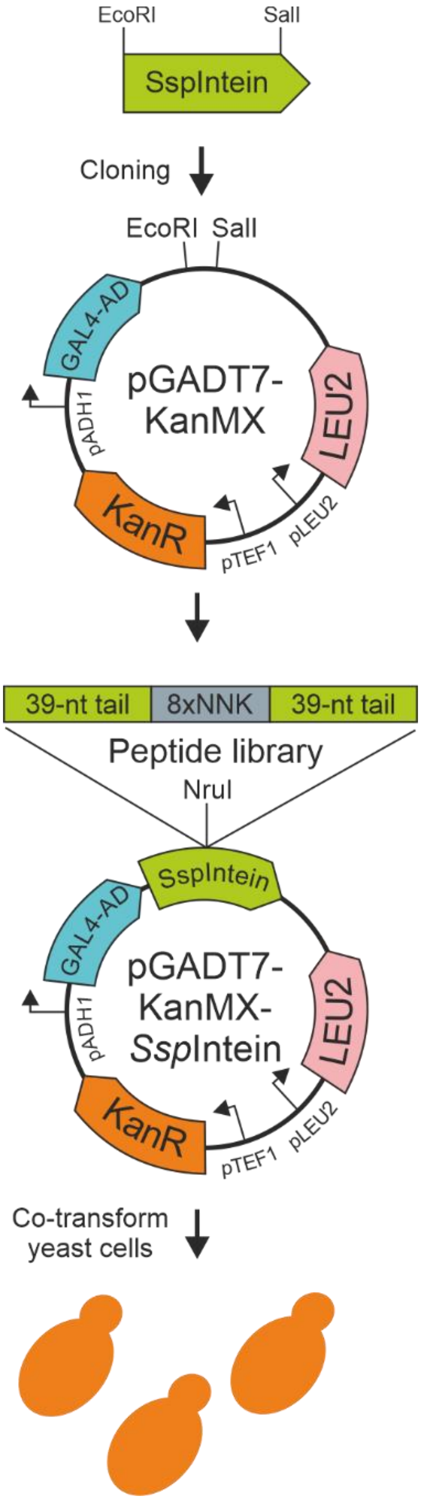

Supplementary Figure S1 – CYCLIC library plasmid construction.

SspIntein encoding gene was cloned into the pGADT7-KanMX vector, downstream to the GAL4-Activation Domain (GAL4-AD). Following cloning, the resulting plasmid (pGADT7-KanMX-SspIntein) was then linearized using the restriction enzyme NruI, to enable insertion of the cyclic peptide library (8xNNK) by *in yeast* homologous recombination.
