# Supplementary material for: The cyclic peptide G4CP2 enables the modulation of galactose metabolism in yeast by interfering with GAL4 transcriptional activity
Source: Front Mol Biosci. 2023 Mar 1;10:1017757. doi: 10.3389/fmolb.2023.1017757 (PMC10014601; doi:10.3389/fmolb.2023.1017757)
Supplement: Supplementary file 11 [file DataSheet5.pdf]

Supplementary Figure S5

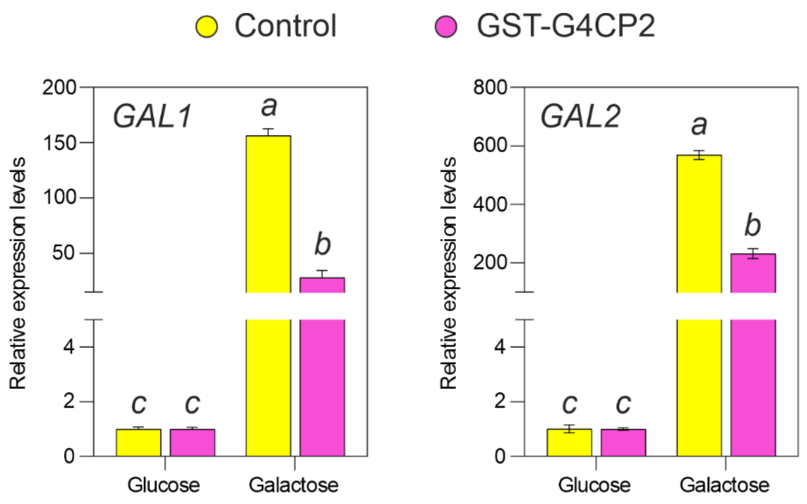

Supplementary Figure S5 – GST-G4CP2 reduces the expression levels of GAL4-activated genes.

Relative expression levels *GAL1* and *GAL2* genes were evaluated after 1 h of incubation under non-inducing conditions (1% glucose) or inducing conditions (1% galactose). The experiment was carried out in BY4741-GAL1p::YFP expressing GST-Intein (Control) or GST-G4CP2. The graph shows that *GAL1* and *GAL2* expression was lower in the strain expressing GST-G4CP2 under under inducing conditions. This result demonstrates that G4CP2 inhibition of GAL4 downstream activity is observable also at the transcriptional level. Statistical significance was determined using One-way ANOVA and represented as compact letter display.
