# Supplementary material for: The cyclic peptide G4CP2 enables the modulation of galactose metabolism in yeast by interfering with GAL4 transcriptional activity
Source: Front Mol Biosci. 2023 Mar 1;10:1017757. doi: 10.3389/fmolb.2023.1017757 (PMC10014601; doi:10.3389/fmolb.2023.1017757)
Supplement: Supplementary file 14 [file DataSheet8.pdf]

Supplementary Figure S8

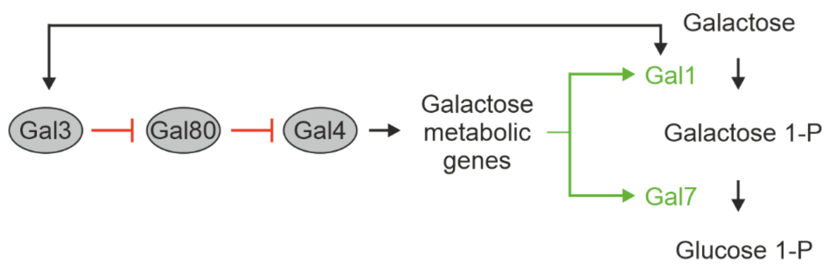

Supplementary Figure S8 – Galactose metabolism regulation in yeast.

When glucose is the main carbon source, the yeast GAL4 transcription factor is repressed at the protein level through an interaction with the GAL80 protein. Upon exposure to increasing levels of galactose, GAL80 inhibition of GAL4 transcriptional activity is relieved by the GAL3 protein, allowing the expression of galactose metabolic genes for galactose metabolization.
