# Supplementary material for: The cyclic peptide G4CP2 enables the modulation of galactose metabolism in yeast by interfering with GAL4 transcriptional activity
Source: Front Mol Biosci. 2023 Mar 1;10:1017757. doi: 10.3389/fmolb.2023.1017757 (PMC10014601; doi:10.3389/fmolb.2023.1017757)
Supplement: Supplementary file 16 [file DataSheet10.pdf]

Supplementary Figure S10

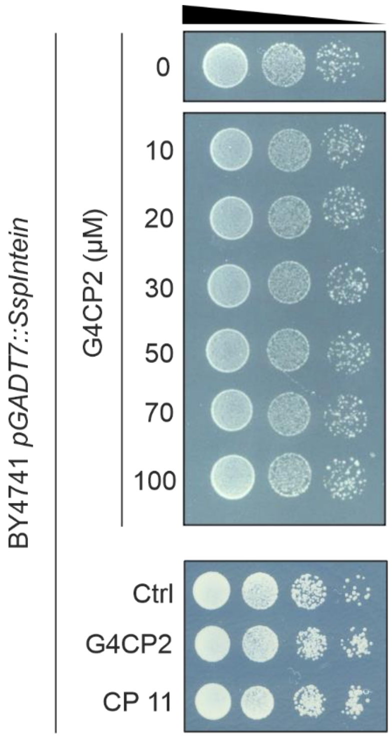

Supplementary Figure S10 – Yeast cells do not show growth defects after G4CP2 treatment.

BY4741 yeast cells expressing the control plasmid (pGADT7-KanMX-SspIntein) were incubated with increasing concentrations of chemically synthesized G4CP2 (from 10 µM to 100 µM) for 8 hours prior to spotting serial dilutions on SD –L +2% glycerol (supplemented with 200 µg/ml of G418). Glycerol was selected as the carbon source as it is neutral to galactose metabolism. As shown, no effects on yeast cell growth were observed. In the lower panel, the same experimental procedure was carried out using synthetic G4CP2 and the CP11 random peptide, CP11 (Figure 4) at 100 µM, displaying no negative effects on yeast growth.
